# Understanding peace through the world news

**DOI:** 10.1140/epjds/s13688-022-00315-z

**Published:** 2022-01-21

**Authors:** Vasiliki Voukelatou, Ioanna Miliou, Fosca Giannotti, Luca Pappalardo

**Affiliations:** 1 Scuola Normale Superiore, Pisa, Italy; 2 Department of Computer & Systems Sciences, Stockholm University, Stockholm, Sweden; 3 Institute of Information Science and Technologies, National Research Council (ISTI-CNR), Pisa, Italy

**Keywords:** Well-being, Peace, News, Global Peace Index, GDELT, Explainable AI, AI for social good, SHAP

## Abstract

Peace is a principal dimension of well-being and is the way out of inequity and violence. Thus, its measurement has drawn the attention of researchers, policymakers, and peacekeepers. During the last years, novel digital data streams have drastically changed the research in this field. The current study exploits information extracted from a new digital database called Global Data on Events, Location, and Tone (GDELT) to capture peace through the Global Peace Index (GPI). Applying predictive machine learning models, we demonstrate that news media attention from GDELT can be used as a proxy for measuring GPI at a monthly level. Additionally, we use explainable AI techniques to obtain the most important variables that drive the predictions. This analysis highlights each country’s profile and provides explanations for the predictions, and particularly for the errors and the events that drive these errors. We believe that digital data exploited by researchers, policymakers, and peacekeepers, with data science tools as powerful as machine learning, could contribute to maximizing the societal benefits and minimizing the risks to peace.

**Supplementary Information:**

The online version contains supplementary material available at 10.1140/epjds/s13688-022-00315-z.

## Introduction

The global challenges regarding people’s well-being in today’s society are manifold. In a major attempt to face them, the Sustainable Development Goals (SDGs) were introduced by the United Nations (UN) Conference on Sustainable Development in Rio de Janeiro in 2012. The objective was to set universal and measurable dimensions to ensure high levels of well-being for everybody. Considering that well-being is a vague and multi-dimensional concept, it cannot be captured as a whole but through a set of health, socio-economic, safety, environmental, and political dimensions [1, 2]. The United Nations Development Programme (UNDP) embodies these dimensions into 17 SDGs such as “Good Health and Well-Being”, “No Poverty”, and “Reduced inequalities” [3–5].

A crucial development is the inclusion of the SDG for “Peace, Justice, and Strong Institutions”, considering that armed violence is on the rise and it is challenging to prevent it [6]. Since 2011, at least 100,000 people have been killed in deadly conflicts, with the majority of them in Afghanistan, Iraq, and Syria. Although the rate of major wars declined over the past decades, the number of civil conflicts and terrorist attacks increased in the last few years, even in developed countries [7].

Governments and the international community often have little warning of abrupt changes in peace and safety, while the war expenses for the war-torn countries weaken their economies. For example, since 1996, the Democratic Republic of Congo has spent on war almost one-third of its gross domestic product [8]. It is hence not surprising that the Expert Panel on Technology and Innovation in UN Peacekeeping recognizes the importance of harnessing the data revolution for the benefit of the international community and peace [9]. In line with the aforementioned, scientific evidence confirms the critical role of AI in accomplishing the SDGs, including the objective for peace [10].

Unfortunately, the use of big data and AI to foster research in the peace and safety field is still at the very beginning [7, 11]. The world’s leading measurement of national peace, i.e., the Global Peace Index (GPI), produced by the Institute for Economics and Peace [12], is captured by institutional surveys and governmental data, which are usually expensive, time-consuming, hard to collect, and could have a lag of up to two or three years [2].

The objective of this study is to demonstrate that a powerful peace index such as GPI [13] can be estimated with the use of AI at a higher time-frequency as compared to the annual GPI score. To tackle this task, we exploit machine learning and news media attention from a digital data source called GDELT [14] as a proxy for estimating and forecasting GPI. News media records generally describe a variety of subject domains (e.g., economic events, political events) and represent a wide range of targets (e.g., opposing politicians) [15]. Considering that GDELT is a free access database updated daily, it can contribute to the monthly estimation of GPI as compared to the real annual GPI. Besides, GPI through GDELT is produced at a low cost and time-efficient way compared to the traditional methodology.

Our results demonstrate that GDELT variables are a good proxy for measuring GPI at a monthly level. In particular, our models exploit the information from GDELT to provide GPI predictions. We perform our analysis for all countries around the world. There are country models that show high performance, such as the United Kingdom and Yemen, countries that show medium performance, such as Chile and Libya, and others that show low performance, such as Estonia and Cyprus. The reasons for the low model’s performance could be various, such as the under- or over-representation of some countries through the GDELT news [16].

In this paper, which expands our previous studies [17, 18], we produce GPI estimates from 1-month-ahead up to 6-months-ahead, conduct the analysis using additional machine learning models, and apply explainable AI techniques to analyze the behavior of high performance models in-depth. Furthermore, we include 12 more recent data points in our analysis, i.e., from April 2019 to March 2020.

To understand better the drivers of the predictions, we use explainable AI techniques [19–21] to identify the relationships between the GDELT variables and peace, and explain the models’ behavior. This analysis allows us to unveil each country’s profile. For example, the most important variables for the United States, such as “Express intent to settle dispute” and “Employ aerial weapons”, indicate a powerful country in military, socio-economic, and political terms. In contrast, the most important variables for Iceland, such as “Praise or endorse” and “Accede to requests or demands for political reform”, denote a peaceful country.

Frequent estimation updates of the GPI score through the GDELT database could flag conflict or war spots months in advance by revealing considerable month-to-month peace fluctuations and significant events that would be otherwise neglected. Consequently, our research could be beneficial to peacekeeping organizations, such as the UN and its agencies, to organize early interventions. In addition, it could be valuable to policymakers to apply adequate policies to prevent detrimental societal effects and contribute effectively to lasting peace.

## Related works

Although peace is a central concept for the global community and peacekeepers strive for its maintenance, it has not a clear definition up to date. Thus, researchers are not easily guided in measuring peace and creating relevant indicators. GPI, the world’s leading measurement of national peace, investigates the extent to which countries are involved in ongoing domestic and international conflicts and seeks to evaluate the level of harmony or discord within a nation. GPI is constructed from 23 indicators that broadly assess what might be described as safety and security in society (detailed list of indicators in Supplementary Note 1 (see Additional file 1)) [12].

Similarly to other peace-related measures [22–24], GPI is captured by official data. Considering the limitations of the official data and the composite index of 23 indicators, it is difficult to have frequent peace updates. Therefore, as conflicts and violence become increasingly complex, policymakers and peacekeepers search for novel approaches to tackle the growing challenge. Big data and AI are potential tools to measure peace-related indicators, produce early warnings of peace changes, and complement estimates from official data.

Social media, such as Twitter, are primarily used to assess public safety, external conflicts, foreign policy, and migration phenomena, as they render individuals’ online activities accessible for analysis. Given this enormous potential, researchers use social media data to predict crime rates or detect the fear of crime [25–28] and to track civil unrest and violent crimes [29–33]. Similarly, Twitter data are used to study early detection of the global terrorist activity [34], military conflicts in Gaza Strip [35, 36], and foreign policy discussions between Israel and Iran [37]. In addition, social media data are useful in estimating turning points in migration trends [38], and stocks of migrants [39, 40]. Finally, researchers have created a French corpus of tweets annotated for event detection, such as conflict, war and peace, crime, and justice [41].

Many researchers use mobility data, such as mobile phone records and GPS traces [42–46] in combination with traditional data, to predict and prevent crime [47–51], compare how the different factors correlate with crime in various cities [52]. Moreover, researchers combine social media data with phone records to infer migration events [53–57] and use GPS data, combined with subjective and objective data, to study perceived safety [58].

Additionally, the volume and momentum of web search queries, such as Google Trends, provide useful indicators of periods of civil unrest over several countries [59, 60], and contribute to capturing a decline in domestic violence calls per capita when immigration enforcement awareness increases [61].

Crowdsourced data are used to map violence against women [62], for police-involved killings [63], for analyzing the international crisis between India and Pakistan for the dispute over Kashmir [64], for preventing crime events, and emergencies [65], and for capturing the fear of crime [66].

Recently, researchers have started exploring remote sensing data, such as satellite images, to map refugee settlements [67, 68] and to study ethnic violence [69], humanitarian crises [70], and conflicts, particularly in zones where field observations are sparse or non-existent [68].

Finally, researchers combine conflict-related news databases such as ACLED [71] with other official data to capture peace indicators and measure conflict risks [72, 73], to demonstrate the relatively short-term decline in conflict events during the COVID-19 pandemic [74], and to create political violence early-warning systems [75]. They also combine the Arabia Inform [76] with official data to extract variables for generating military event forecasts [77].

GDELT is a major news data source that describes the worldwide socio-economic and political situation through the eyes of the news media, making it ideal for measuring well-being and peace [14]. GDELT is mainly used to explore social unrest, protests, civil wars and coups, crime, migration, and refugee patterns. Many researchers explain and predict social unrest events in several geographic areas around the world, such as in Egypt [78], Southeast Asia [79], the United States [80], and Saudi Arabia [81]. Other researchers recognize social unrest patterns in India, Pakistan, and Bangladesh [82], and reveal the causes and evolution of future social unrest events in Thailand [83]. GDELT is a valuable source of data for the detection of protest events [84] and violence-related social issues [85], as well as for detecting and forecasting domestic political crises [86]. It is also used for the exploration of severe internal and external conflicts, such as the Sri Lankan civil war, the 2006 Fijian coup [87], and the Afghanistan violence events [88]. Additionally, it helps in understanding the direct cooperative and conflictual interactions among China, Russia, and the US since the end of the Cold War [89]. Also, GDELT is used to study activities of political nature influencing or reflecting societal-scale behavior and beliefs [90]. Lastly, news data from GDELT are combined with other data sources, such as socio-economic indicators [91], refugee data [92], and housing market data [93], Google Trends, and official migration data [94], to analyze and produce short and medium-term forecasts of migration patterns.

Our paper differs from previous work in two important aspects. First, our models harness GDELT with machine learning techniques to estimate a composite peace index as GPI, covering domestic and international conflicts, safety and security, migration phenomena, etc. The wide variety of GDELT event categories can cover most GPI indicators. Second, we perform our analysis at a global scale to study peace over all countries in the world.

## Methodology

This section describes the data used in our study, the models used to produce the GPI estimates, the training strategy adopted, and the SHAP methodology applied to interpret the models’ predictions. We provide the data and the code of our study for reproducibility in https://github.com/VickyVouk/GDELT_GPI_SHAP_project [95].

### GPI data

GPI [13] ranks 163 independent states and territories according to their level of peace, and it was created by the Institute for Economics & Peace (IEP). GPI data are available from 2008 until 2020 at a yearly level (GPI report 2020 [12]). The score for each country is continuous, normalized on a scale of 1 to 5, where the higher the score, the less peaceful a country is. For example, in 2019, Iceland was the most peaceful country with $\mathrm{GPI} =1.072$, whereas Somalia was the least peaceful country with $\mathrm{GPI} =3.574$. The index is constructed from 23 indicators related to Ongoing Domestic and International Conflict, Societal Safety and Security, and Militarisation domains [12] (detailed list of indicators in Supplementary Note 1 (see Additional file 1)). These indicators are weighted and combined into one overall score. The weights for the GPI indicators can be retrieved from the GPI reports [12]. For the GPI construction, data are derived from official sources, such as governmental data, institutional surveys, and military data.

For this study, we increase GPI frequency from yearly to monthly data using linear interpolation. Every yearly GPI value is assigned to March of the corresponding year since most of the annual GPI indicators are measured until this month. The linear upsampling is the simplest assumption since the monthly data generated do not correspond to the real monthly GPI. After upsampling, from 13 yearly values, we obtain 145 months in total (March 2008–March 2020).

We increase the frequency from yearly to monthly data because a month might contain important events distorted from the yearly index. Indeed, the yearly GPI data might not indicate abrupt peace changes at a higher frequency because they are smoothed out on the yearly GPI value. Therefore, monthly GPI estimations could reveal events neglected from the yearly GPI. At the same time, we do not increase the frequency at a weekly or daily level to keep a trade-off between the noisy weekly or daily GDELT information and the official yearly GPI. Besides, daily or weekly estimates could indicate fluctuations that would not significantly change a country’s stability for weeks or even months after taking place.

**Figure 1:**
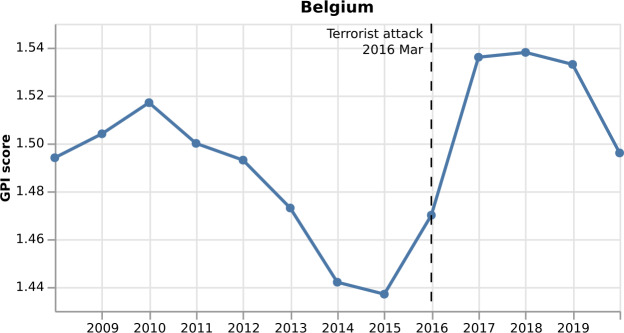
*Monthly GPI for Belgium from 2008 to 2020*. In March 2016, the terrorist attack took place in Belgium, and as a result, the GPI increases

**Figure 2:**
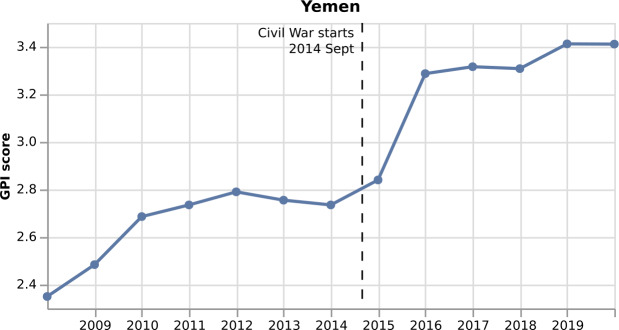
*Monthly GPI for Yemen from 2008 to 2020*. In September 2014, the Civil War started in Yemen, and as a result, the GPI increases

Figure 1 and Fig. 2 show the monthly GPI for Belgium and Yemen, respectively, from 2008 to 2020. In Fig. 1, we annotate the terrorist attack that took place in Belgium in March 2016, which brought a deterioration in the peace level of the country, increasing GPI from 1.47 to 1.536. However, this is depicted in the real yearly GPI only a year later, in 2017. On the contrary, when we introduce the monthly GPI score, we expect our model to depict the increase more timely, e.g., one month after the attack.

In Fig. 2, we annotate the start of the Civil War in Yemen in September 2014, which brings a deterioration in the country’s peace level, increasing GPI from 2.735 to 2.84. Since the real GPI is only published once a year, it seems that the increase starts from March 2014, i.e., six months before the actual event. With the monthly GPI score, we expect our model to capture this change in the GPI one month after the start of the Civil War.

As a consequence, a monthly system that adequately corresponds to the peace fluctuations has the potential to quickly inform the placement of peacekeepers and the deployment of non-governmental organization (NGO) resources, making it potentially easier to save lives and prevent devastation [75].

### GDELT data

GDELT [14] is a Google-supported and publicly available digital news database related to socio-political events. It is a collection of international English-language news sources, such as the Associated Press and The New York Times. GDELT data are based on news reports coded with the Tabari system [96], which extracts the events from the media and assigns the corresponding code to each event. Events are coded based on an expanded version of the dyadic CAMEO format, a conflict, and mediation event taxonomy [97]. GDELT compiles a list of 200 categories of events, from riots and protests to peace appeals and diplomatic exchanges, from public statements and consulting to fights and mass violence [97] (detailed list of topics in Supplementary Note 2 (see Additional file 1)). Examples of identified events are “Express intent to cooperate”, “Conduct strike or boycott”, “Use conventional military force”, and “Reduce or break diplomatic relations”.

The database offers various information for each event, such as the date, location, and the URL of the news article. We use GDELT 1.0 database, which is updated daily and contains historical data since 1979 [98].

For GPI prediction, we derive several variables from GDELT, corresponding to the total number of events (No. events) of each GDELT category at a country and monthly level. Some event categories may not be present in the news of a country. On average, the number of variables per country is 87, varying from 25 to 141. We use the BigQuery [99] data manipulation language in the Google Cloud Platform to extract the GDELT variables (Listing 1). 
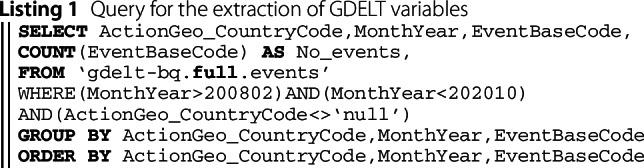


**Figure 3:**
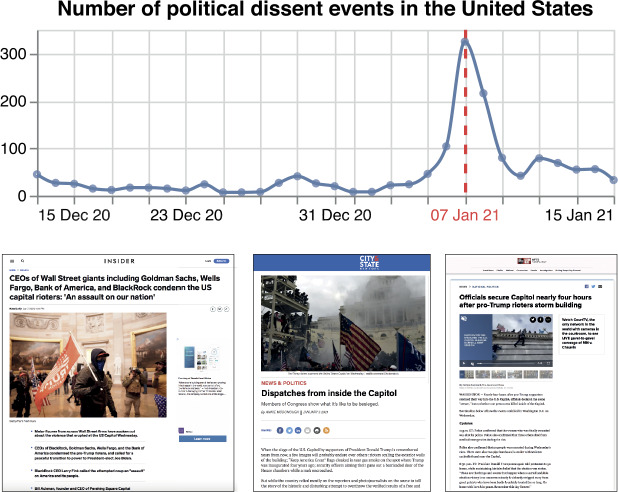
*Number of political dissent events in the United States*. Daily number of political dissent events (blue curve) derived from the GDELT news in the United States, from the middle of December 2020 to the middle of January 2021, and three news articles published on the 6th and 7th of January. GDELT depicts a noticeable rise of the events related to political dissent on the 6th of January 2021, the day of the “Storming of the United States Capitol”, and a peak of news related to the topic on the 7th of January 2021 (vertical dashed red line)

In Fig. 3, we present an example of the number of events related to engagement in political dissents, such as civilian demonstrations, derived from the GDELT news on the United States, from the middle of December 2020 to the middle of January 2021. We also present three news articles published on the 6th and 7th of January. The plot depicts a noticeable rise in these events on the 6th of January 2021, the day of the “Storming of the United States Capitol”, and a peak of news related to the topic on the 7th of January 2021, showing how GDELT news depicts the worldwide sociopolitical and conflictual reality with a small lag, i.e., a day.

Table 1 shows some GDELT records for the United States in February and March 2018. For example, in February 2018, the No. events for the category “Investigate crime” is 680, and in March 2018, it is 799. In February 2018, the No. events for the category “Conduct non-military bombing” is 523, and in March 2018, it is 1099. The latter variable’s value has increased a lot from February to March 2018 because of the “Austin serial bombings” (five package bombs exploded) that occurred between the 2nd of March and 22nd of 2018, mainly in Austin, Texas.

**Table 1:** Examples of the United States variables in February and March 2018. The event code and category that describe the event are reported. The No. events that occurred are also presented

Event code	Event category	No. events	Date
⋮	⋮	⋮	⋮
022	Appeal for diplomatic cooperation	2168	2018/02
091	Investigate crime	680	2018/02
122	Reject, request or demand for material aid	501	2018/02
183	Conduct non-military bombing	523	2018/02
⋮	⋮	⋮	⋮
022	Appeal for diplomatic cooperation	2561	2018/03
091	Investigate crime	799	2018/03
122	Reject, request or demand for material aid	534	2018/03
183	Conduct non-military bombing	1099	2018/03
⋮	⋮	⋮	⋮

Table 2 presents the 10 GDELT variables with the largest share of No. events for the United States from March 2008 to March 2020. For example, the GDELT variable “Make statement” has the largest share, followed by “Make a visit” and “Host a visit” variables.

**Table 2:** The ten GDELT variables with the largest share of the number of news for the United States, i.e., from March 2008 to March 2020

Event code	Event category	Share over all news
010	Make statement	7.73 %
042	Make a visit	7.52 %
043	Host a visit	6.97 %
020	Make an appeal or request	6.61 %
051	Praise or endorse	5.80 %
040	Consult	5.59 %
036	Express intent to meet or negotiate	4.50 %
173	Arrest, detain, or charge with legal action	4.08 %
190	Use conventional military force	3.72 %
046	Engage in negotiation	2.85 %

### Matching GPI indicators with GDELT variables

The wide variety of GDELT event categories covers most GPI indicators. For example, the GPI indicator “Number of Internal Security Officers and Police per 100,000 People” can be covered by the GDELT variable “Exhibit military or police power”. The GPI indicators “Ease of Access to Small Arms and Light Weapons” and “Volume of Transfers of Major Conventional Weapons, as recipient (imports) per 100,000 people” can be covered by “Fight with small arms and light weapons” and “Use conventional military force” or “Conduct non-military bombing” GDELT variables, respectively. Similarly, the “Nuclear and Heavy Weapons Capabilities” GPI indicator can be covered by the “Employ aerial weapons” GDELT variable. Also, the GPI indicator “Likelihood of violent demonstrations” can be covered by “Engage in political dissent”, “Protest violently, riot” or “Demonstrate or rally” GDELT variables. Last, the “Financial Contribution to UN Peacekeeping Missions” GPI indicator can be covered by the GDELT variables “Appeal for aid” or “Provide humanitarian aid”.

### Predictive models

Models handling time series are used to predict future values of indices by extracting relevant information from historical data. Traditional time series models are based on various mathematical approaches, such as autoregression. Autoregressive models specify that the output variable depends linearly on its previous values and a stochastic term. Considering that our data are upsampled linearly, it is not feasible to apply autoregressive models because of the linear relationship between the dependent variable (GPI) and its past values. Besides, our objective is to measure GPI and understand and explain how different peace topics captured by GDELT contribute to the GPI measurement.

We use Linear Regression, Elastic Net, Decision Tree, Support Vector Regression (SVR), Random Forest, and Extreme Gradient Boosting (XGBoost) to investigate the relationship between the GPI score and the GDELT variables at a country level. Specifically, we aim to develop GPI estimates 1-month-ahead to 6-months-ahead of the latest ground-truth GPI value and find the model with the highest performance overall. Firstly, we introduce simple models, i.e., Linear Regression, Elastic Net, and Decision Tree, which are easy to implement and interpret. Next, we apply SVR, Random Forest, and XGBoost models, which tend to achieve higher predictive performance but are harder to interpret, and they need additional methodologies for the interpretation of the results (e.g., SHAP [20, 21]). Our main goal is to find the model with the highest predictive performance. Supplementary Note 3 (see Additional file 1) briefly describes the characteristics of the selected models.

### Estimation framework

Before modeling, researchers start by dividing the data into training and test data. Training data are used to estimate the models’ parameters, and the test data are used to calculate the predictive performance of the models.

Considering that the socio-political situation around the world is not stationary and more recent events are relevant for the prediction, we train our models using the rolling methodology [100], widely used in business and finance [101]. The rolling methodology updates the training set by an add/drop process while keeping its length stable and retrains the model before each *k*-months-ahead prediction.

The rolling training’s set period for all models is half of our data, i.e., 72 months. First, we train the model to predict from 1-month-ahead to 6-months-ahead GPI values. After the first training, one month is dropped from the beginning of the training set, and another month is added to the end of the training set. Then, we perform the training again to predict the next 1-month-ahead to 6-months-ahead GPI values. We continue this training process for all subsequent months until we predict the last monthly value. This process ensures that the training set covers the same amount of time and is continuously updated with the most recent information.

In particular, we use data from March 2008 to February 2014 (72 values) to train the model and predict the GPI values of March 2014 up to August 2014, data from April 2008 to March 2014 (72 values) to train the model and predict the GPI values of April 2014 up to September 2014, and so on. We repeat this procedure until the last training, which includes data from March 2014 to February 2020 (72 values), to make a 1-month-ahead GPI prediction, corresponding to March 2020, the last time series value.

We obtain from 1-month-ahead up to 6-months-ahead predicted GPI values at each step. Specifically, by the end of each rolling training described above, we have *k*-months-ahead GPI predictions, where $k=1,2,\dots ,6$ months. By the end of the training process, we have 72 1-month-ahead GPI predictions,^1^ 71 2-months-ahead GPI predictions, and so on. We evaluate the accuracy of the predictions for each *k*-months ahead time horizon to the corresponding test set that contains the real GPI values. Long-term predictions, such as 6-months-ahead peace estimations, are an important tool for policymakers since it is a “policy-relevant lead time” consistent with other forecasting work; that is, a period sufficiently long that there could be a policy response [102].

For each of the models mentioned in Sect. 3.4, we estimate the best hyperparameters in each training phase through 10-fold cross-validation. Supplementary Note 4 (see Additional file 1) includes all the details for the hyperparameters we tune for each model, except for Linear regression, which has no hyperparameters.

### Model interpretation through SHAP

Understanding a model’s prediction is important for trust, actionability, accountability, debugging, and many other reasons. To understand predictions from tree-based machine learning models, like Random Forest or XGBoost, importance values are typically attributed to each variable. Yet traditional variable attribution for trees is inconsistent, meaning it can lower a variable’s assigned importance when the true impact of that variable increases.

Therefore, for the interpretation of the importance of the model variables and for understanding the drivers of every single GPI estimation, we compute the SHAP (SHapley Additive exPlanation) values [20, 21]. SHAP is based on game theory [103], and local explanations [104], and it offers a means to estimate the contribution of each variable. By focusing specifically on tree-based models, the authors developed an algorithm that computes local explanations based on exact Shapley values in polynomial time. SHAP provides local explanations with theoretical guarantees of local accuracy and consistency. Additionally, the ability to efficiently compute local explanations using Shapley values over a dataset enables the development of a range of tools to interpret and understand a model’s global behavior. Specifically, by combining many local explanations, a global structure can be represented while retaining local faithfulness [105] to the original model, which generates detailed and accurate representations of the model’s behavior.

Last but not least, SHAP can be applied to interpret the results of the machine learning models since it identifies the relationship between the independent variables, either internal or external and the dependent variable. The relationship between the independent and dependent variables does not need to be causal, as SHAP could fail to answer causal questions accurately. In this study, SHAP is a tool to identify which external GDELT variables drive the GPI estimations. This can be useful for explaining the models’ behavior and diagnosing errors in the predictions.

## Results

The predictive models introduced in Sect. 3.4 are constructed for every country using the GPI values as the dependent variable and the GDELT variables as the independent variables. We use the Pearson Correlation coefficient, Root Mean Square Error (RMSE), and Mean Absolute Percentage Error (MAPE) [106–108] to evaluate the performance of the constructed models (Supplementary Note 5 (see Additional file 1)).

**Figure 4:**
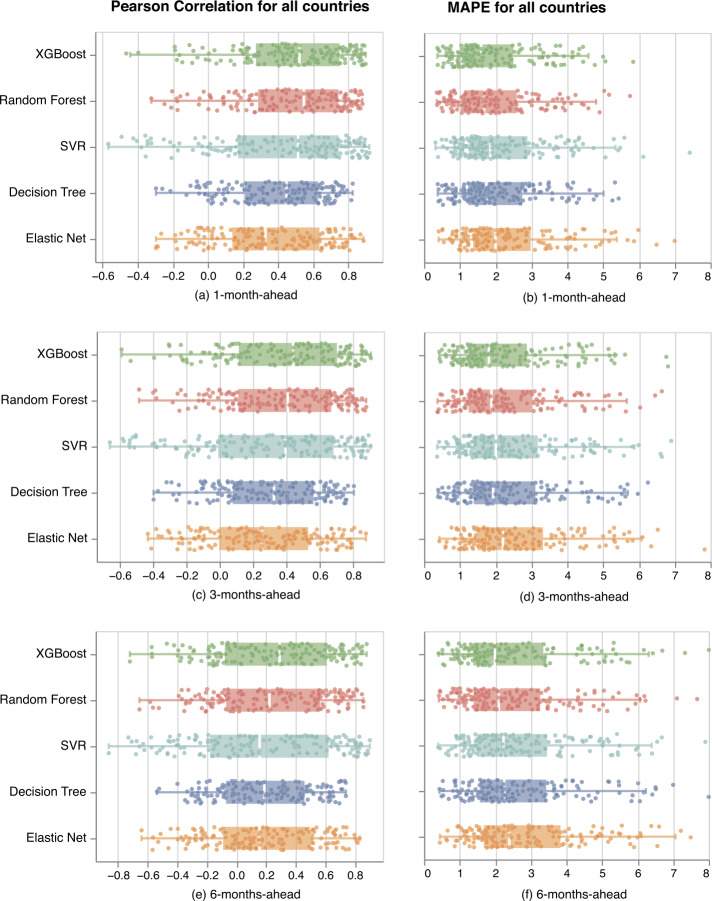
*Pearson Correlation and MAPE for all country models*. Pearson Correlation and MAPE between the real and the predicted 1-, 3-, and 6-months-ahead GPI values at a country level for all predictive models. The boxplots represent the distribution of the Pearson correlation and MAPE for all country models. The plots’ data points correspond to each country model. Overall, XGBoost outperforms all other models

The analysis is conducted for all 163 countries with a GPI score, and we generate 1-month-ahead up to 6-months-ahead predictions. Figure 4 presents Pearson Correlation and MAPE between the real and the 1-, 3-, and 6-months-ahead predicted GPI values at a country level for all predictive models.^2^ Figure 1 in Supplementary Note 7 (see Additional file 1) presents the RMSE performance indicator as well. We find that SVR, Random Forest, and XGBoost have similar performance and outperform Decision Tree and Elastic Net. XGBoost shows the highest performance overall, especially for the 6-months-ahead predictions.

For the estimation of the GPI, the models use the historical data of the No. events for each GDELT category related to the military, social, and political events of the corresponding country. For each additional future estimation, we move further away from the last training data while the country’s reality changes, and we, therefore, expect a lower model performance. Indeed, comparing Figs. 4(a)–(b), with Figs. 4(c)–(d), and with Figs. 4(e)–(f), we show that the performance of the models decreases for every additional month-ahead prediction. For example, median MAPE increases by 13.43% for the 3-months-ahead predictions and by 25.61% for the 6-months-ahead predictions, compared to the 1-month-ahead predictions.

**Figure 5:**
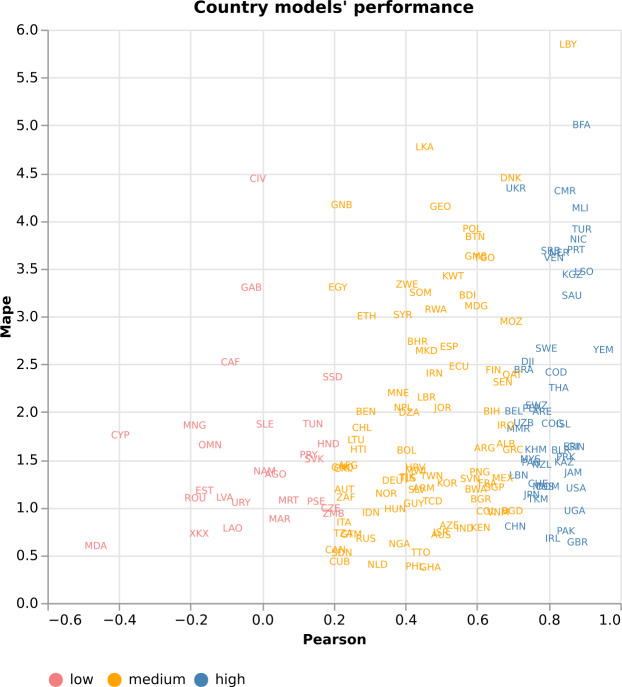
*High, medium, and low performance country models*. High, medium, and low performance country models for the 1-month-ahead predictions. There are country models that show high performance, such as the United Kingdom (GBR), models that show medium performance, such as Libya (LBY), and models that show low performance, such as Mongolia (MNG)

Since XGBoost achieves the highest performance overall and produces good predictions for either low, medium, or high GPI values (Fig. 2 in Supplementary Note 8 (see Additional file 1)), we focus on it when presenting the subsequent results. We divide the countries into three categories based on their performance. We consider high performance models those with $\mbox{Pearson Correlation} \geq 0.7$ and $\mathrm{MAPE}< 5$ [109, 110], low performance models those with $\mbox{Pearson Correlation} \leq 0.2$ [110], and the rest of the models are considered medium performance models. Figure 5 presents the countries with high, medium, and low performance for the 1-month-ahead predictions. For example, Uganda (UGA), Pakistan (PAK), Turkey (TUR), the United Kingdom (GBR), and Sweden (SWE) show high-performance, with a strong Pearson Correlation, higher than 0.8. We also observe medium performance countries, such as Libya (LYB) with high Pearson Correlation but high MAPE, and India (IND) with low Pearson Correlation but low MAPE. Finally, there are countries, such as Cyprus (CYP), Estonia (EST), Moldova (MDA), Mongolia (MNG), and Romania (ROU), which show a negative Pearson Correlation.

### High performance models

Our study aims to demonstrate that GDELT is a valuable digital news data source for estimating the GPI at a monthly level. For this reason, we present the performance indicators and analyze in-depth the models that confirm this hypothesis, i.e., the country models with high performance. Since conflicts and violence are present in every country, despite it being in war or not, we present countries with different military, socio-economic, and political histories and current situations to cover a variety of scenarios.

In particular, we present three of the most powerful countries (United States, United Kingdom, and Saudi Arabia) since they shape global economic patterns and influence policymaking [111]. Additionally, we use various sources, such as the official GPI ranking [12], to choose three of the most peaceful countries (Portugal, Iceland, and New Zealand) and three of the most war-torn countries (DR Congo, Pakistan, and Yemen).

Table 3 reports the models’ performance for the 1-month-ahead up to 6-months-ahead GPI estimates for nine countries. Overall, 1-month-ahead GPI estimates are more accurate than the other estimates, especially to the 6-months-ahead estimates. There are countries, such as Portugal, for which the performance remains stable overall 6 months predictions and countries like Yemen for which the performance falls for each additional in future prediction.

**Table 3:** Performance indicators with respect to GPI ground-truth of nine high performance country models. Overall, 1-month-ahead GPI estimates are significantly more accurate compared to the rest future estimates, especially to the 6-months-ahead time horizon

Countries	Performance indicators	Prediction framework						Mean
		1-month-ahead	2-months-ahead	3-months-ahead	4-months-ahead	5-months-ahead	6-months-ahead	
United States	Pearson	0.876	0.838	0.813	0.782	0.750	0.710	0.795
	MAPE (%)	1.197	1.367	1.465	1.592	1.700	1.899	1.537
	RMSE	0.037	0.040	0.042	0.045	0.048	0.053	0.044
United Kingdom	Pearson	0.880	0.849	0.848	0.845	0.853	0.850	0.854
	MAPE (%)	0.632	0.742	0.787	0.821	0.826	0.981	0.798
	RMSE	0.015	0.017	0.017	0.018	0.018	0.020	0.017
Saudi Arabia	Pearson	0.864	0.848	0.849	0.814	0.772	0.781	0.822
	MAPE (%)	3.213	3.406	3.733	4.126	4.396	4.590	3.911
	RMSE	0.089	0.094	0.101	0.111	0.119	0.123	0.106
Portugal	Pearson	0.876	0.868	0.868	0.838	0.835	0.820	0.851
	MAPE (%)	3.691	4.241	4.539	5.221	5.067	5.538	4.716
	RMSE	0.057	0.065	0.067	0.077	0.075	0.080	0.070
Iceland	Pearson	0.840	0.833	0.827	0.810	0.770	0.731	0.802
	MAPE (%)	1.867	2.014	2.114	2.256	2.283	2.367	2.150
	RMSE	0.025	0.027	0.028	0.030	0.030	0.031	0.028
New Zealand	Pearson	0.780	0.748	0.725	0.692	0.689	0.650	0.714
	MAPE (%)	1.444	1.538	1.633	1.651	1.741	1.793	1.633
	RMSE	0.023	0.024	0.025	0.026	0.026	0.027	0.025
DR Congo	Pearson	0.820	0.815	0.790	0.762	0.740	0.728	0.776
	MAPE (%)	2.409	2.792	2.856	2.899	2.957	3.120	2.839
	RMSE	0.088	0.099	0.103	0.105	0.107	0.113	0.103
Pakistan	Pearson	0.848	0.772	0.720	0.668	0.672	0.640	0.720
	MAPE (%)	0.749	0.858	0.922	1.006	1.052	1.036	0.937
	RMSE	0.029	0.033	0.036	0.040	0.040	0.040	0.036
Yemen	Pearson	0.832	0.771	0.746	0.722	0.687	0.662	0.737
	MAPE (%)	5.063	6.033	6.810	7.287	7.801	7.999	6.832
	RMSE	0.207	0.243	0.267	0.283	0.300	0.309	0.268
Yemen^∗^	Pearson	0.953	0.945	0.934	0.922	0.908	0.898	0.892
	MAPE (%)	2.645	2.990	3.440	3.652	3.914	4.171	4.287
	RMSE	0.116	0.129	0.144	0.154	0.166	0.176	0.180

^∗^For the training of this model, the most recent 36 monthly values are used, as compared with the rest of the countries’ models that are trained with the most recent 72 monthly values.

An explanation for these different behaviors could be, for example, in the case of Portugal, that the military, socio-economic, and political situation remains stable over time. Therefore the most important variables contribute to a more accurate prediction even further in the future. On the contrary, in war-torn countries like Yemen, the country’s situation changes constantly, and the variables are not much relevant anymore. For this reason, for Yemen, we also conduct training with the 36 most recent monthly values (Yemen^∗^ in Table 3), as opposed to the 72 values used for the rest of the countries. The performance improves considerably: the mean Pearson Correlation increases from 0.737 to 0.892, the mean MAPE drops from 6.832 to 4.287, and the mean RMSE decreases from 0.268 to 0.180. However, we do not observe the same improvement in the performance when decreasing the training set for the other war-torn countries, such as DR Congo.

Furthermore, we select four countries to study in-depth their peace and the factors that drive it. We aim to capture various scenarios on the models’ accuracy and the models’ explanation. Particularly, we choose Saudi Arabia and Yemen to understand better and interpret the results and errors of the predictive models based on historical data. Additionally, we choose the United Kingdom and the United States to estimate their future GPI values to gain initial insights into the country’s peace before the official GPI score becomes available.

#### Saudi Arabia

Based on the G20 list of countries [111], Saudi Arabia is considered one of the most powerful countries in the world in terms of military and international alliances, political and economic influence, and leadership.

**Figure 6:**
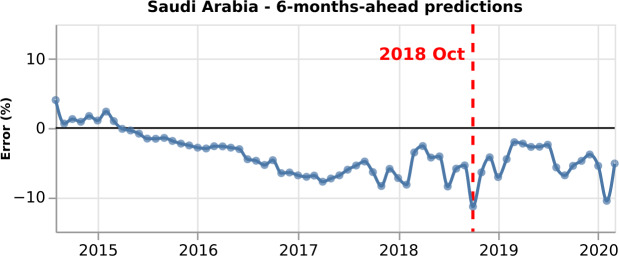
*Percentage error for Saudi Arabia*. Percentage error for the 6-months-ahead GPI estimations (blue curve). The performance is very high, and the percentage error varies, in absolute values, from 4.05% to 11.38%. We obtain the largest negative percentage error for the GPI estimation of October 2018 (vertical dashed red line)

Figure 6 presents the percentage error of Saudi Arabia for the 6-months-ahead GPI estimations. We observe high performance, and the percentage error varies from 4.05% to 11.38%. A positive percentage error indicates that the estimated GPI is higher than the real GPI, and therefore the model overestimates the monthly value. On the contrary, a negative percentage error illustrates that the estimated GPI is lower than the real GPI, and thus the model underestimates the monthly value. We obtain the largest negative percentage error for the GPI estimation of October 2018.

**Figure 7:**
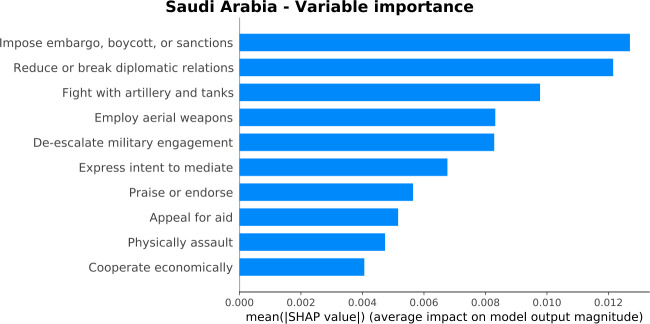
*Variable importance plot for Saudi Arabia*. The barplot orders the variables based on their importance in estimating the GPI score. The most important variables demonstrate a profile of a powerful country in military, socio-economic, and political terms

The analysis of the variable importance through SHAP reveals the country’s profile and helps us understand the larger errors of the model. Figure 7 shows the most important variables for the estimation of the GPI score. Each importance is calculated by combining many local explanations, and the model is trained between May 2012 to April 2018. The important variables reveal the profile of a powerful country in military, socio-economic and political terms. Indeed, they are related to embargo, boycott, or sanctions, diplomatic relations, mediations, economic cooperations, and appeals for aid, fights with military arms, military engagement, assaults, and endorsements. In Fig. 7, we also observe that “Fight with artillery and tanks” and “Appeal for aid” are among the most important variables for Saudi Arabia. As discussed in Sect. 3.3, these GDELT variables could cover the “Volume of Transfers of Major Conventional Weapons, as recipient (imports) per 100,000 people” and the “Financial Contribution to UN Peacekeeping Missions” GPI indicators, respectively.

**Figure 8:**
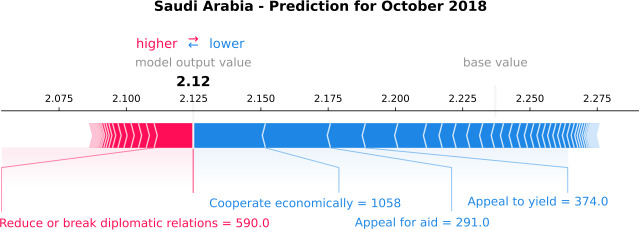
*Individual SHAP Value plot for Saudi Arabia*. It presents the model output value, i.e., the estimation of the GPI of October 2018, and the base value, which is the value that would be predicted if the variables for the current output were unavailable. The plot also displays the most important variables that the model uses for the GPI estimation, such as “Cooperate economically” and “Appeal for aid”. The red arrows are the variables that push the GPI estimation higher, and the blue ones push the estimation lower

To explain better why the model has the worst performance in October 2018, we perform SHAP analysis at a local level to highlight the most important variables that the model uses for this specific estimation. Figure 8 displays the most important variables that Saudi Arabia’s model uses for the GPI estimation of October 2018. The model output value is 2.12, corresponding to the 6-months-ahead prediction. The base value is higher than the estimated GPI, and it is the value that would be predicted if the variables for the current output were unavailable. The red arrows are the variables that push the GPI estimation higher (to the right), and those blue push the estimation lower (to the left). Considering that this month the model underestimates the GPI value (Fig. 6), we focus on the variables that push the GPI estimation lower.

**Figure 9:**
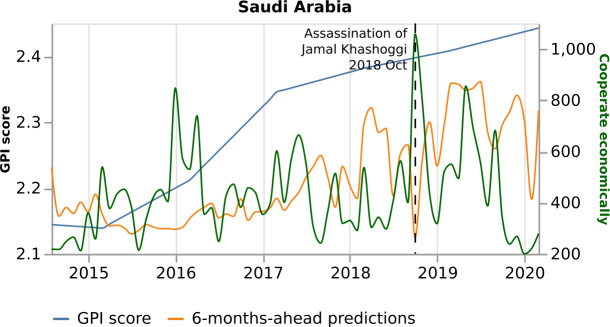
*Saudi Arabia predictions, with respect to the real GPI score, and the variable “Cooperate economically”*. Saudi Arabia 6-months-ahead predictions (orange curve), with respect to the real GPI score (blue curve), and the variable “Cooperate economically” (green curve). This variable pushes the model to underestimate the monthly value of October 2018 (vertical dashed black line). The reason for this error is the assassination of Jamal Khashoggi in this specific month

**Figure 10:**
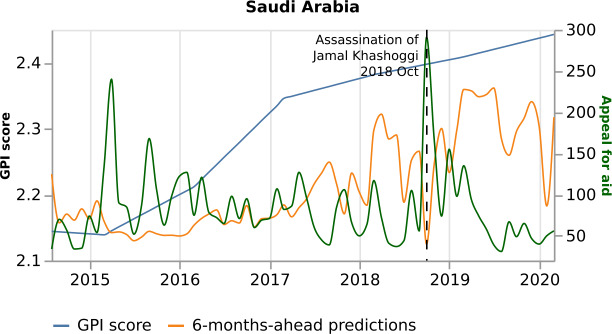
*Saudi Arabia predictions, with respect to the real GPI score, and the variable “Appeal for aid”*. Saudi Arabia predictions (orange curve), with respect to the real GPI score (blue curve), and the variable “Appeal for aid” (green curve). This variable pushes the model to underestimate the monthly value of October 2018 (vertical dashed black line). The reason for this error is the assassination of Jamal Khashoggi in this specific month

The most important variables for the prediction of October 2018 are “Cooperate economically” and “Appeal for aid”, although they are 10th and 8th respectively in the model’s overall ranking of importance (Fig. 7). In October 2018, the journalist Jamal Khashoggi was assassinated at the Saudi consulate in Istanbul, Turkey. This event provoked a series of news on the topics mentioned above. Figure 9 presents Saudi Arabia’s model predictions to the real GPI score and the variable “Cooperate economically”. This variable shows an abrupt increase in October 2018 and pushes GPI prediction lower, showing a more peaceful month. Similarly, Fig. 10 shows an abrupt increase of the variable “Appeal for aid” in October 2018 and drives the prediction lower, showing a more peaceful month. Considering that the assassination of the journalist is a negative event, one would expect a less peaceful month. However, looking at the news, the articles discuss possible spills into oil markets and economic cooperation between Saudi Arabia and other countries, such as the United States, to overcome a dispute over Khashoggi. In addition, the news is also concentrated on the investigation of the Khashoggi case, such as Amnesty International asking for a UN inquiry. Therefore, considering that the variables “Cooperate economically”, and “Appeal for aid” have a negative relationship with GPI (Figs. 9 and 10, respectively) the model underestimates the monthly value. Consequently, through the eyes of the world news, the presentation of peace is not always at the level we would expect.

#### Yemen

Based on the official GPI ranking [13], Yemen is one of the most war-torn countries in the world. Hence, it is interesting to understand the model’s behavior for such a country’s profile in-depth.

The situation in Yemen constantly changes due to the Civilian War that broke out in September 2014. The change of peace in the country is depicted in the real GPI value, which abruptly increases in 2015 [13]. Therefore, six years of training data related to the pre-war period would not be representative for the model to predict peace after the beginning of the war since the No. events related to the military, economic, and political situation of the country changes. Thus, we decrease the training set to the most recent three years. We use the rolling methodology to throw the pre-war historical data more quickly and learn from the most recent and relevant data related to the post-war period. Therefore, for Yemen, we use data from March 2015 to March 2020 to understand the model’s behavior during the Civil War period.

**Figure 11:**
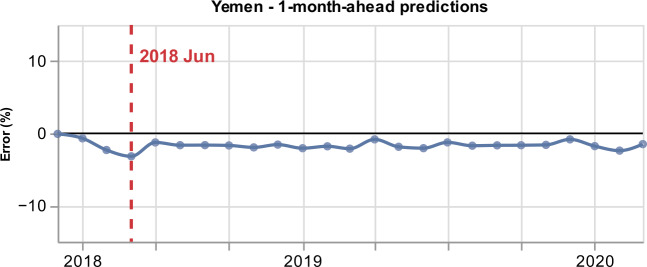
*Percentage error for Yemen*. Percentage error for the 1-month-ahead GPI estimations (blue curve). The percentage error varies, in absolute values, from 0.07% to 3.18%. We obtain the largest negative percentage error for the GPI estimation of June 2018 (vertical dashed red line)

Figure 11 presents the percentage error for 1-month-ahead GPI estimations from March 2018 to March 2020 with a training period of 36 months. The model has a high performance, with a low percentage error that varies from 0.07% to 3.18% with a median value of 1.66%. We obtain the largest negative percentage error (underestimation of GPI) in June 2018.

**Figure 12:**
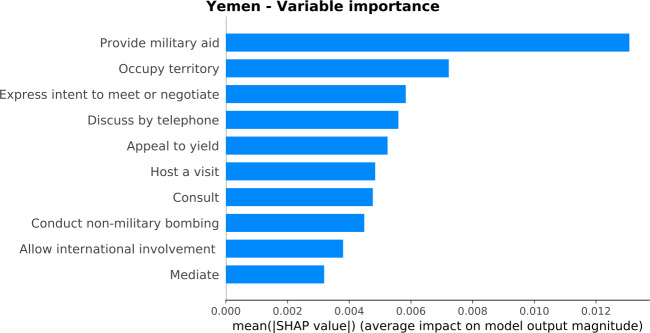
*Variable importance plot for Yemen*. The barplot orders the variables based on their importance in estimating the GPI score. The most important variables demonstrate a country with a war-torn profile

Figure 12 displays the most important variables for the estimation of the GPI. Each variable importance is calculated through SHAP, with a training period from June 2015 to May 2018. Overall, the most important variables reveal a war-torn country profile since they are related to military aid, territory occupation, bombing, negotiations, discussions, yields, visits, international involvements, and consults. In Fig. 12, “Conduct non-military bombing” is among the most important variables. As discussed in Sect. 3.3, this GDELT variable could cover the “Volume of Transfers of Major Conventional Weapons” GPI indicator.

**Figure 13:**
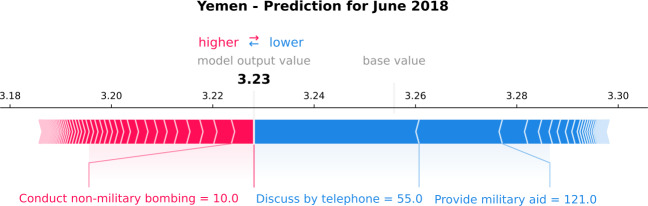
*Individual SHAP Value plot for Yemen*. It presents the model output value, i.e., the GPI estimation of June 2018, and the base value, which is the value that would be predicted if the variables for the current output were unavailable. The plot also displays the most important variables that the model uses for the GPI estimation, such as “Discuss by telephone” and “Provide military aid”. The red arrows are the variables that push the GPI estimation higher, and the blue ones push the estimation lower

Similarly to Saudi Arabia, we analyze at a local level to understand why the model produces the highest percentage error in June 2018. Figure 13 displays the variables that drive the prediction of June 2018. The model output value is 3.23, which corresponds to the 1-month-ahead prediction. The red arrows represent the variables that push the GPI estimation higher, i.e., “Conduct non-military bombing”. The blue arrows represent the variables that push the GPI estimation lower, i.e., “Discuss by telephone” and “Provide military aid”. Considering that in June 2018, the model underestimates the monthly value (Fig. 11), we focus on the latter variables.

In June 2018, the number of events on “Discuss by telephone” is 55, higher than the median value (14) of the previous three years. Similarly, the number of events on “Provide military aid” is 121, higher than the median value (72) of the previous three years. In June 2018, the United Arab Emirates Armed Forces (UAE) announced a pause to the military operations on the 23rd of June 2018 because of UN-brokered talks. This is depicted in the news increase on “Discuss by telephone”. In addition, the United States turned down UAE requests for aid in the offensive against rebel-held Yemeni port, thanks to the UN efforts. This denial has been discussed a lot in the media, which explains the increase of the news on “Provide military aid”.

**Figure 14:**
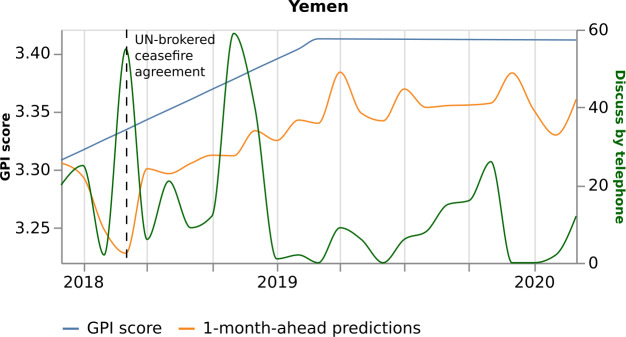
*Yemen predictions, with respect to the real GPI score and the variable “Discuss by telephone”*. Yemen 1-month-ahead predictions (orange curve), with respect to the real GPI score (blue curve) and the variable “Discuss by telephone” (green curve). This variable pushes the model to underestimate the GPI value of June 2018 (vertical dashed black line). The reason for this error is the increase of the news on the topic in this specific month

**Figure 15:**
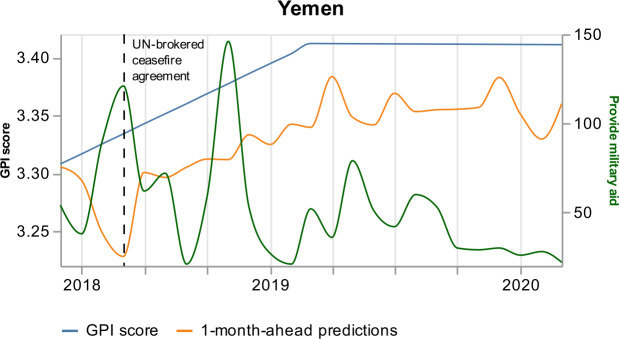
*Yemen predictions, with respect to the real GPI score and the variable “Provide military aid”*. Yemen 1-month-ahead model predictions (orange curve), with respect to the real GPI score (blue curve) and the variable “Provide military aid” (green curve). This variable pushes the model to underestimate the GPI value in June 2018 (vertical dashed black line). The reason for this error is the increase of the news on the topic in this specific month

Figures 14 and 15 show that the variables’ higher monthly value and their mostly negative relationship with the GPI drive the model to underestimate the GPI value in June 2018. Consequently, June 2018 results more peaceful than it was. On the one hand, the model makes a wrong prediction, resulting in the largest percentage error. On the other hand, the model might give an interesting signal. Although Yemen is involved in constant conflicts, June 2018 results more peaceful since the UN-brokered ceasefire agreement managed the withdrawal of the warring parties from Al Hudaydah in Yemen. Last, although we notice additional abrupt increases of the two variables’ values, e.g., in November 2018 (Figs. 14 and 15), the model does not reproduce an abrupt decrease of the GPI. Thus, the model shows its power to learn from its mistakes.

#### United States

The United States is considered the most powerful country in the world [111]. On that account, it is interesting to study its peace after March 2020. The United States model shows a high performance (Table 3) and can provide policymakers and peacekeepers with valuable initial insights into the country’s peace before the real GPI score becomes available.

**Figure 16:**
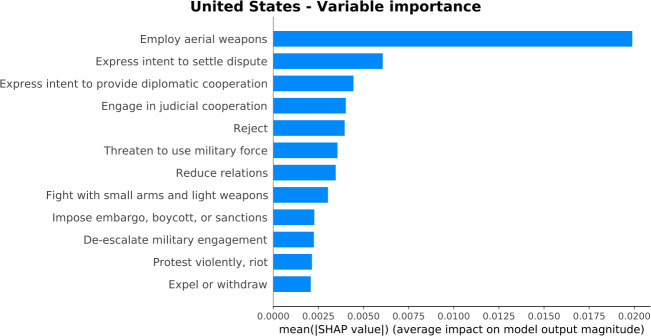
*Variable importance plot for the United States*. The barplot orders the variables based on their importance in estimating the GPI score. The most important variables indicate a country profile of a strong player in the military, socio-economic, and political foreground

To start with, Fig. 16 shows the most important variables for the training period between April 2014 and March 2020. Overall, these variables indicate a country profile of a strong player in the military, socio-economic, and political foreground. The most important variable is related to aerial weapons, and it mainly concerns events that take place overseas. Additionally, the rest of the variables are mostly related to fights with small arms, military de-escalations, embargoes, threats, protests, cooperations, and relations. We also observe in Fig. 16 that “Employ aerial weapons”, “Fight with small arms and light weapons”, and “Protest violently, riot” are among the most important variables for the United States. As discussed in Sect. 3.3, these GDELT variables could correspond to GPI indicators “Nuclear and Heavy Weapons Capabilities”, “Ease of Access to Small Arms and Light Weapons”, and “Likelihood of violent demonstrations”, respectively. Last, we compare the variables in Fig. 16 with the ten variables that have the largest share of overall news (Table 2 in Sect. 3.2). None of the variables that have the largest share of overall news is among the most important variables for the United States. This confirms that the model is unbiased to learn only from the variables with the largest share. It selects the variables that adequately serve for making the peace prediction. In other words, even if there is an event that attracts most news attention, what matters for the model is the relationship between the GPI and each variable’s time-series.

**Figure 17:**
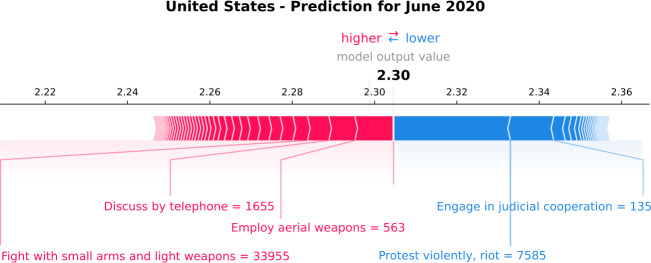
*Individual SHAP Value plot for the United States*. It presents the model output value, i.e., the GPI estimation of June 2020. The plot also displays the most important variables that the model uses for the GPI estimation, such as “Protest violently, riot”. The red arrows are the variables that push the GPI estimation higher, and the blue ones push the estimation lower

We now focus on the murder of George Floyd, which took place on the 25th of May, 2020. Several protests followed this event at the end of May and for the whole of June 2020, provoking news concentrated on the topic. Figure 17 shows the local SHAP explanation for the prediction of June 2020. The estimated GPI (3-months-ahead prediction) is 2.30, indicating that the GPI value will remain high in June 2020 compared with the last ground-truth value of March 2020 (2.31) and the median GPI value of the previous three years (2.34). Mainly, “Protest violently, riot” is the variable that pushes the GPI estimation lower. Indeed, in June 2020, the news was concentrated on a series of protests, followed by the murder of George Floyd against police brutality and racism. This variable pushes for a more peaceful month since it has a negative relationship with the GPI. It seems that protesting in the United States contributes to improving various socio-political situations and peacekeeping.

The rest of the variables displayed in Fig. 17 have lower values than their corresponding median values of the training period, confirming that the news of the month was concentrated on the United States racial unrest and the Black Lives Matter movement. We point out that, in this particular prediction, the most important variable for the overall training period, i.e., “Employ aerial weapons” (Fig. 16), has a less important contribution to the model output as compared with the variable “Protest violently, riot”. This proves the power of SHAP in identifying the role of each variable for every single prediction.

#### United Kingdom

Similar to the United States and Saudi Arabia, based on the list of G20 [111], the United Kingdom is considered one of the most powerful countries in the world. It is hence interesting for the European social policymaking to anticipate the level of peace after the last ground-truth data, i.e., after March 2020.

**Figure 18:**
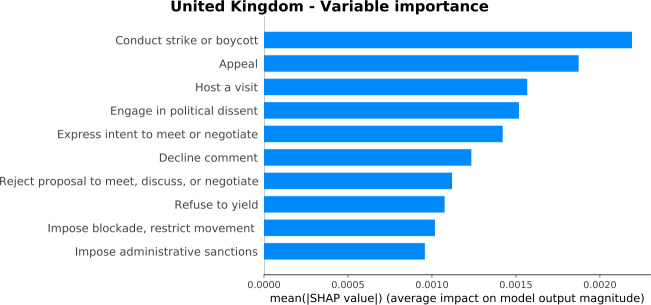
*Variable importance plot for the United Kingdom*. The barplot orders the variables based on their importance in estimating the GPI score. The most important variables demonstrate a country where various socio-political events occur

We focus on the GPI prediction of July 2020, where various restrictions related to Covid-19 and the civilians’ protection were announced. Figure 18 presents the variable importance plot for a training period from April 2014 to March 2020. The figure highlights a country where various socio-political events occur since the important variables are mostly related to strikes or boycotts, appeals, negotiations, yields, relationships, and sanctions. “Engage in political dissent” is among the most important variables for the United Kingdom (Fig. 18). As discussed in Sect. 3.3, this variable could cover the GPI indicator “Likelihood of violent demonstrations”.

**Figure 19:**
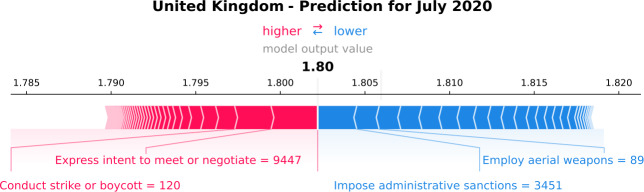
*Individual SHAP Value plot for the United Kingdom*. It presents the model output value, i.e., the estimation of the GPI of July 2020. The plot also displays the most important variables that the model uses for the GPI estimation, such as “Express intent to meet or negotiate” and “Conduct strike or boycott”. The red arrows are the variables that push the GPI estimation higher, and the blue ones push the estimation lower

To study peace in July 2020, we need to deepen the analysis at a local level. Figure 19 presents the individual SHAP value plot for the United Kingdom. The GPI value is 1.8, and it is the model output value for the 4-months-ahead prediction. The GPI in July 2020 is slightly higher than the last ground-truth value (1.77), and it is stable compared to the median GPI value of the previous three years (1.8).

The most important variables that push the GPI value higher are “Express intent to meet or negotiate” and “Conduct strike or boycott”. The former variable’s value is 9447, which is lower than the median value of the previous six years (12,026). The latter variable’s value is 120, slightly lower than the median value of the previous six years (126). These results show that lower values of these event categories decrease internal peace in the United Kingdom. The value decrease of these event categories could be due to the COVID-19 restrictions or the news concentrated on the COVID-19 pandemic. Additionally, “Impose administrative sanctions” and “Employ aerial weapons” are the variables that drive the GPI prediction lower. The former’s value in July 2020 is 3451, higher than the variable’s median value of the previous six years (2590). The news related to “Impose administrative sanctions” concern discussions on restrictions due to the pandemic, despite the easing of the lockdown. Furthermore, many articles discuss the ban to Huawei from the 5G network due to security risks and the ban on junk food advertising and promotion in-store. Consequently, the model has learned that although “Impose administrative sanctions” events restrict people, the deeper aim of the restrictions is to protect them. Last, the “Employ aerial weapons” variable’s value is 89, much lower than the median value of the previous six years (167), pushing the GPI value lower. This variable is referred to overseas events that the United Kingdom is involved. The decrease in its value might demonstrate that the news does not discuss it due to previous de-escalations or because the news is concentrated on other topics.

### Medium and low performance countries

There are country models which demonstrate medium performance (Sect. 4 and Fig. 5), such as Colombia and Chile ($\mbox{Pearson Correlation} = 0.63$ and $\mathrm{MAPE} = 0.96$, and $\mbox{Pearson Correlation} = 0.28$ and $\mathrm{MAPE} = 1.83$, respectively, for the 1-month-ahead predictions). To get insights into the reasons behind the medium performance, we further study these country models.

**Figure 20:**
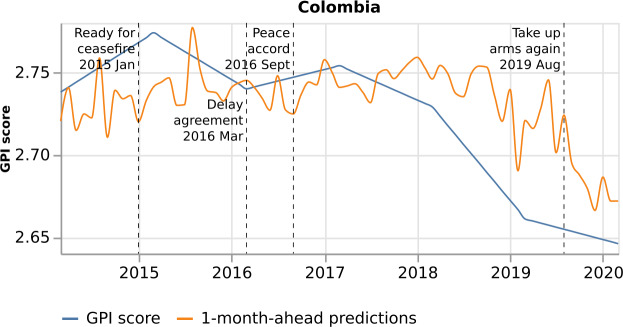
*Colombia predictions, with respect to the real GPI score*. Colombia 1-month-ahead predictions (orange curve), with respect to the real GPI score (blue curve). The estimated GPI adequately captures the changes in peace in January 2015, March 2016, September 2016, and August 2019, compared to the real GPI

Colombia ranks 11th out of 163 countries on the list presenting the economic cost of violence ranked by percentage of GDP. Particularly, its economic cost of violence is 169,517 (in million 2019 PPP U.S. dollars) [12]. Thus, in line with the study’s purposes, it would be important to understand and explain why the model shows a medium performance. Figure 20 presents Colombia’s model predictions to the real GPI score. Colombia has been pursuing peace since 1964. Therefore we focus on a selected sample of important events to show how well our model captures peace fluctuations and why predictions may vary compared to the real GPI score.

In January 2015, President Santos said the government was ready for a bilateral ceasefire with Farc after welcoming Farc’s December unilateral ceasefire. The estimated GPI captures the decrease of GPI, as opposed to the real GPI that continues increasing. In March 2016, the government and Farc delayed signing a final agreement. In this case, the estimated GPI adequately captures the GPI increase compared to the real GPI that decreases. Similarly, in September 2016, the government and Farc signed a historic peace accord. Thus, the estimated GPI is correctly decreased this month, compared to the real GPI that continues increasing. Last, in August 2019, the Farc rebel group commander defied the 2016 peace agreement and called on supporters to take up arms again. Consequently, the GPI score should increase, and Colombia’s model adequately captures this peace fluctuation compared to the real GPI that continues decreasing. The real GPI score does not depict these peace changes because it is a monthly index upsampled from a yearly index. Therefore, some small changes are smoothed out on the real index or if important ones are depicted later on the following year (Sect. 3.1 includes further details on the upsampled GPI).

**Figure 21:**
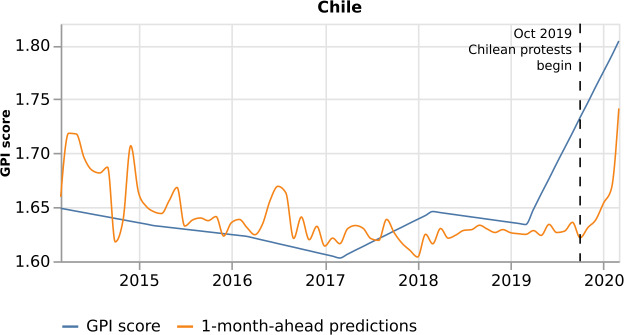
*Chile predictions, with respect to the real GPI score*. Chile 1-month-ahead predictions (orange curve), with respect to the real GPI score (blue curve). The estimated GPI score adequately captures the disturbance in peace in October 2019, when the Chilean protests began, compared to the real GPI score

In addition to Colombia, we analyze Chile to understand its medium performance better. Based on the 2020 GPI report [12], Chile has its lowest levels of peace since the inception of the GPI. Figure 21 depicts Chile’s model predictions to the real GPI score. The plot demonstrates that the predictions curve follows the real GPI curve till March 2019. In March 2019, we observe the real GPI increasing abruptly till March 2020, and the predictions curve does not follow the real GPI till October 2019. In October 2019, Chile was rocked by mass protests at economic inequality, prompted by a subsequently-reversed rise in Santiago metro fares. The estimated GPI score, in contrast with the real GPI score, captures this increase on time. The real GPI might anticipate this increase because it is a monthly index upsampled from a yearly index. Therefore it depicts the abrupt peace turbulence already from March 2019.

We also deepen the analysis to find out why some country models show low performance. To control to what extent these countries are covered from the GDELT news, we investigate if there is any correlation between each country’s mean number of the overall news and model’s performance or between each country’s mean number of monthly news and the model’s monthly performance. However, we do not find out any correlation. Another possible explanation for some countries’ low performance, which could be further explored, is that some countries might be under-represented through the GDELT news or even over-represented [16]. For example, many United States news media, which is the strongest player in the media industry, are tracked by GDELT. The United States news in the English language might not sufficiently cover events happening in foreign countries or non-English speaking countries.

Moreover, news media could introduce additional biases in the study. First, they sometimes misrepresent reality. For example, they give a distorted version of the crimes within a city with a significant bias towards violence [112]. Second, news media datasets contain the gatekeeping bias, i.e., the journalists decide on which event to publish, the coverage bias, related to the over-coverage or under-coverage of an event, and the statement bias, i.e., when the content of an article might be favorable or unfavorable towards certain events [113].

## Conclusion

New technologies have been increasingly acknowledged as critical tools to foster peace [114, 115]. In particular, new digital data streams harnessed with AI allow for predictive analytics to enhance early warning about emerging conflicts and operational risks, cost- and time-effectively.

We exploit GDELT, a digital news database related to socio-political events, to estimate the monthly peace values through GPI. Measuring the GPI score at a monthly level indicates trends at a much finer scale than it is possible with the yearly official measurements, capturing fluctuations and significant events that would be otherwise neglected. We use machine learning to estimate the GPI values from 1-month-ahead up to 6-months-ahead for 163 countries worldwide, with different socio-economic, political, and military profiles. There are country models that show high performance, while others show medium or low performance. We conduct in-depth analysis on country models with high performance, such as Saudi Arabia, Yemen, the United States, and the United Kingdom. We also apply explainable AI techniques to provide explanations for the models’ results and reveal the profile of each country. For example, the most important variables for Yemen are related to military aid, territory occupation, bombing, negotiations, discussions, yields, visits, international involvements, and consults, revealing a war-torn country profile. Additionally, we use explainable AI techniques to provide explanations for the predictions of specific months for the selected countries. Explainable AI techniques allows us to explain the errors in the predictions and identify the events that drive these errors.

There is an aspect of our study that we should take into consideration. Since GPI is a yearly index, we upsample its yearly values linearly to monthly values. The linear upsampling is an assumption since the monthly data generated do not correspond to the real monthly GPI. Alternatively, another assumption could be to increase the frequency of GPI through stochastic differential equation (SDE) methods [116], a more complex methodology than simple linear interpolation. Considering that both solutions are assumptions and that our main goal is to demonstrate that monthly peace can be captured through the news data, we choose the simplest one. Future studies could deepen more the analysis by trying different upsampling methodologies. An alternative solution could be replacing GPI with a monthly index, which would not require upsampling.

Another line of future research lies in analysing the results per country. Indeed, for certain countries the models show low performance in predicting the GPI. One approach to improve the models’ performance is to change the training data length based on the history of the country, usually depicted on the GPI. For example, as we show for Yemen, the performance improves by changing the training data from the most recent 72 months to the most recent 36 months.

Additionally, news media might introduce biases, driving the models to show low performance in predicting the GPI value. Therefore, it would be beneficial to study in-depth the representativeness of GDELT news, as some countries might be under-represented or over-represented, to help us explain why some models fail to demonstrate high or at least medium performance.

Last but not least, we highlight that machine learning models are a powerful tool for solving prediction problems. Still, they are not inherently causal, and interpreting them with techniques like SHAP fails to answer causal questions accurately. Therefore, we indicate two additional points that can improve early-warning conflict systems: first, more information about the causes of conflicts and war and, second, theoretical models representing the complexity of social interactions and human decision-making. In particular, future AI-based conflict models should offer explanations for conflicts and war and plans for preventing them. This is a difficult task because conflict and war dynamics are multi-dimensional, and the data collected today are too narrow, sparse, and disparate [7].

Overall, the analysis of our results shows great promise for the estimation of GPI through GDELT and, in general, for the measurement of peace using big data and AI. Our study is valuable to policymakers, peacekeepers, the scientific community, and especially to researchers interested in “Data Science for Social Good”. Indeed, GDELT could be used not only for peace but for any other well-being dimension and socio-economic index related to societal progress.

## Supplementary Information

Below is the link to the electronic supplementary material.

Supplementary information (PDF 6.1 MB)

## Data Availability

The code to reproduce the study is available at https://github.com/VickyVouk/GDELT_GPI_SHAP_project [95].

## Footnotes

1 According to the initial test set’s length.

2 Since the Linear model has very low performance (Supplementary Note 6 (see Additional file 1)), we present the results for all models but the Linear regression.

## References

[1] Organisation for Economic Co-operation and Development (2011) How’s life?: measuring well-being. OECD, Paris

[2] Voukelatou V, Gabrielli L, Miliou I, Cresci S, Sharma R, Tesconi M, Pappalardo L (2021). Measuring objective and subjective well-being: dimensions and data sources. Int J Data Sci Anal.

[3] UNDP (2015) Sustainable development goals. https://sustainabledevelopment.un.org/sdgs

[4] Kroll C, Warchold A, Pradhan P (2019). Sustainable development goals (SDGs): are we successful in turning trade-offs into synergies?. Palgrave Commun.

[5] Le Blanc D (2015). Towards integration at last? The sustainable development goals as a network of targets. Sustain Dev.

[6] Bank W (2018) Pathways for peace: inclusive approaches to preventing violent conflict. The World Bank

[7] Guo W, Gleditsch K, Wilson A (2018). Retool AI to forecast and limit wars. Nature.

[8] Hillier D (2007) Africa’s missing billions: international arms flows and the cost of conflict

[9] Perera S (2017). To boldly know: knowledge, peacekeeping and remote data gathering in conflict-affected states. Int Peacekeeping.

[10] Vinuesa R, Azizpour H, Leite I, Balaam M, Dignum V, Domisch S, Felländer A, Langhans SD, Tegmark M, Nerini FF (2020). The role of artificial intelligence in achieving the sustainable development goals. Nat Commun.

[11] Wählisch M (2020). Big data, new technologies, and sustainable peace: challenges and opportunities for the UN. J Peacebuilding Dev.

[12] The Institute for Economics and Peace (2020) Global Peace Index 2020

[13] The Institute for Economics and Peace (2017) Vision of humanity. http://visionofhumanity.org//

[14] Leetaru K (2013) The GDELT project. https://www.gdeltproject.org/

[15] Balahur A, Steinberger R, Kabadjov M, Zavarella V, Van Der Goot E, Halkia M, Pouliquen B, Belyaeva J (2013) Sentiment analysis in the news. arXiv preprint. arXiv:1309.6202

[16] Kwak H, An J (2014). A first look at global news coverage of disasters by using the GDELT dataset. International conference on social informatics.

[17] Voukelatou V, Pappalardo L, Miliou I, Gabrielli L, Giannotti F (2020). Estimating countries’ peace index through the lens of the world news as monitored by GDELT. 2020 IEEE 7th international conference on data science and advanced analytics (DSAA).

[18] Voukelatou V, Miliou I, Pappalardo L (2021) Stima dell’indice di pace attraverso notizie digitali. Lettura ragionata dell’Enciclica Papale “Fratelli tutti”, alla luce dell’Obiettivo 16 dell’Agenda Onu 2030, 57–60

[19] Guidotti R, Monreale A, Ruggieri S, Turini F, Giannotti F, Pedreschi D (2019). A survey of methods for explaining black box models. ACM Comput Surv.

[20] Lundberg SM, Erion GG, Lee S-I (2018) Consistent individualized feature attribution for tree ensembles. arXiv preprint. arXiv:1802.03888

[21] Lundberg S, Lee S-I (2017) A unified approach to interpreting model predictions. arXiv preprint. arXiv:1705.07874

[22] Brückner M, Ciccone A (2010). International commodity prices, growth and the outbreak of civil war in sub-Saharan Africa. Econ J.

[23] Gries P, Fox A, Jing Y, Mader M, Scotto TJ, Reifler J (2020). A new measure of the ‘democratic peace’: what country feeling thermometer data can teach us about the drivers of American and Western European foreign policy. Politl Res Exch.

[24] The Institute for Economics and Peace (2011) Structures of peace: identifying what leads to peaceful societies

[25] Chen X, Cho Y, Jang SY (2015). Crime prediction using Twitter sentiment and weather. 2015 systems and information engineering design symposium.

[26] Al Boni M, Gerber MS (2016). Predicting crime with routine activity patterns inferred from social media. 2016 IEEE international conference on systems, man, and cybernetics (SMC).

[27] Kadar C, Brüngger RR, Pletikosa I (2017). Measuring ambient population from location-based social networks to describe urban crime. International conference on social informatics.

[28] Curiel RP, Cresci S, Muntean CI, Bishop SR (2020). Crime and its fear in social media. Palgrave Commun.

[29] Chen F, Neill DB (2014). Non-parametric scan statistics for event detection and forecasting in heterogeneous social media graphs. Proc. of the 20th ACM SIGKDD international conference on knowledge discovery and data mining.

[30] Nobles M, Neill DB, Flaxman S (2014) Predicting and preventing emerging outbreaks of crime

[31] Neill DB, Gorr WL (2007). Detecting and preventing emerging epidemics of crime. Adv Dis Surveill.

[32] Tucker R, O’Brien DT, Ciomek A, Castro E, Wang Q, Phillips NE (2021). Who ‘tweets’ where and when, and how does it help understand crime rates at places? Measuring the presence of tourists and commuters in ambient populations. J Quant Criminol.

[33] Spangler E, Smith B (2021). Let them tweet cake: estimating public dissent using Twitter. Def Peace Econ.

[34] Najjar E, Al-Augby S (2021). Sentiment analysis combination in terrorist detection on Twitter: a brief survey of approaches and techniques. Research in intelligent and computing in engineering.

[35] Zeitzoff T (2011). Using social media to measure conflict dynamics: an application to the 2008–2009 Gaza conflict. J Confl Resolut.

[36] Siapera E, Hunt G, Lynn T (2015). # GazaUnderAttack: Twitter, Palestine and diffused war. Inf Commun Soc.

[37] Zeitzoff T, Kelly J, Lotan G (2015). Using social media to measure foreign policy dynamics: an empirical analysis of the Iranian–Israeli confrontation (2012–13). J Peace Res.

[38] Zagheni E, Garimella VRK, Weber I, State B (2014). Inferring international and internal migration patterns from Twitter data. Proc. of the 23rd international conference on world wide web.

[39] Zagheni E, Weber I, Gummadi K (2017) Leveraging Facebook’s advertising platform to monitor stocks of migrants. Popul Dev Rev, 721–734

[40] Alexander M, Polimis K, Zagheni E (2020). Combining social media and survey data to nowcast migrant stocks in the United States. Popul Res Policy Rev.

[41] Mazoyer B, Cagé J, Hervé N, Hudelot C (2020). A French corpus for event detection on Twitter. Proceedings of the 12th language resources and evaluation conference.

[42] Toch E, Lerner B, Ben-Zion E, Ben-Gal I (2019). Analyzing large-scale human mobility data: a survey of machine learning methods and applications. Knowl Inf Syst.

[43] Pappalardo L, Simini F, Barlacchi G, Pellungrini R (2021) Scikit-mobility: a Python library for the analysis, generation and risk assessment of mobility data. arXiv preprint. arXiv:1907.07062

[44] Blondel VD, Decuyper A, Krings G (2015). A survey of results on mobile phone datasets analysis. EPJ Data Sci.

[45] Andrienko G, Andrienko N, Boldrini C, Caldarelli G, Cintia P, Cresci S, Facchini A, Giannotti F, Gionis A, Guidotti R (2021). (So) big data and the transformation of the city. Int J Data Sci Anal.

[46] Luca M, Barlacchi G, Lepri B, Pappalardo L (2021). A survey on deep learning for human mobility. ACM Comput Surv.

[47] Bogomolov A, Lepri B, Staiano J, Oliver N, Pianesi F, Pentland A (2014). Once upon a crime: towards crime prediction from demographics and mobile data. Proc. of the 16th international conference on multimodal interaction.

[48] Ariel B, Partridge H (2017). Predictable policing: measuring the crime control benefits of hotspots policing at bus stops. J Quant Criminol.

[49] Ferrara E, De Meo P, Catanese S, Fiumara G (2014). Detecting criminal organizations in mobile phone networks. Expert Syst Appl.

[50] Robinson AI, Carnes F, Oreskovic NM (2016). Spatial analysis of crime incidence and adolescent physical activity. Prev Med.

[51] Wu J, Frias-Martinez E, Frias-Martinez V (2020). Addressing under-reporting to enhance fairness and accuracy in mobility-based crime prediction. Proceedings of the 28th international conference on advances in geographic information systems.

[52] De Nadai M, Xu Y, Letouzé E, González MC, Lepri B (2020). Socio-economic, built environment, and mobility conditions associated with crime: a study of multiple cities. Sci Rep.

[53] Chi G, Lin F, Chi G, Blumenstock J (2020). A general approach to detecting migration events in digital trace data. PLoS ONE.

[54] Sîrbu A, Andrienko G, Andrienko N, Boldrini C, Conti M, Giannotti F, Guidotti R, Bertoli S, Kim J, Muntean CI (2021). Human migration: the big data perspective. Int J Data Sci Anal.

[55] Hankaew S, Phithakkitnukoon S, Demissie MG, Kattan L, Smoreda Z, Ratti C (2019). Inferring and modeling migration flows using mobile phone network data. IEEE Access.

[56] Lai S, zu Erbach-Schoenberg E, Pezzulo C, Ruktanonchai NW, Sorichetta A, Steele J, Li T, Dooley CA, Tatem AJ (2019). Exploring the use of mobile phone data for national migration statistics. Palgrave Commun.

[57] Deville P, Linard C, Martin S, Gilbert M, Stevens FR, Gaughan AE, Blondel VD, Tatem AJ (2014). Dynamic population mapping using mobile phone data. Proc Natl Acad Sci.

[58] DaViera AL, Roy AL, Uriostegui M, Fiesta D (2020). Safe spaces embedded in dangerous contexts: how Chicago youth navigate daily life and demonstrate resilience in high-crime neighborhoods. Am J Community Psychol.

[59] Qi H, Manrique P, Johnson D, Restrepo E, Johnson NF (2016). Open source data reveals connection between online and on-street protest activity. EPJ Data Sci.

[60] Qi H, Manrique P, Johnson D, Restrepo E, Johnson NF (2016). Association between volume and momentum of online searches and real-world collective unrest. Results Phys.

[61] Muchow AN, Amuedo-Dorantes C (2020). Immigration enforcement awareness and community engagement with police: evidence from domestic violence calls in Los Angeles. J Urban Econ.

[62] Lea SG, D’Silva E, Asok A (2017). Women’s strategies addressing sexual harassment and assault on public buses: an analysis of crowdsourced data. Crime Prev Community Saf.

[63] Ozkan T, Worrall JL, Zettler H (2018). Validating media-driven and crowdsourced police shooting data: a research note. J Crime Justice.

[64] Palakodety S, KhudaBukhsh AR, Carbonell JG (2019) Hope speech detection: a computational analysis of the voice of peace. arXiv preprint. arXiv:1909.12940

[65] Rumi SK, Shao W, Salim FD (2020). Realtime predictive patrolling and routing with mobility and emergency calls data. Proceedings of the international AAAI conference on web and social media.

[66] Solymosi R, Buil-Gil D, Vozmediano L, Guedes IS (2021). Towards a place-based measure of fear of crime: a systematic review of app-based and crowdsourcing approaches. Environ Behav.

[67] Quinn JA, Nyhan MM, Navarro C, Coluccia D, Bromley L, Luengo-Oroz M (2018). Humanitarian applications of machine learning with remote-sensing data: review and case study in refugee settlement mapping. Philos Trans R Soc A, Math Phys Eng Sci.

[68] Witmer FD (2015). Remote sensing of violent conflict: eyes from above. Int J Remote Sens.

[69] Marx A, Loboda T (2013). Landsat-based early warning system to detect the destruction of villages in Darfur, Sudan. Remote Sens Environ.

[70] Li X, Li D (2014). Can night-time light images play a role in evaluating the syrian crisis?. Int J Remote Sens.

[71] Clionadh R, Linke A, Hegre H, Karlsen J (2010). Introducing ACLED-armed conflict location and event data. J Peace Res.

[72] Brauer J, Anderton CH (2020). Conflict and peace economics: retrospective and prospective reflections on concepts, theories, and data. Def Peace Econ.

[73] Firchow P, Ginty RM (2017). Measuring peace: comparability, commensurability, and complementarity using bottom-up indicators. Int Stud Rev.

[74] Ide T (2021). Covid-19 and armed conflict. World Dev.

[75] Hegre H, Allansson M, Basedau M, Colaresi M, Croicu M, Fjelde H, Hoyles F, Hultman L, Högbladh S, Jansen R (2019). Views: a political violence early-warning system. J Peace Res.

[76] Inform A (1998) Arabia inform. http://arabiainform.com/

[77] Hossain KT, Gao S, Kennedy B, Galstyan A, Natarajan P (2020). Forecasting violent events in the middle East and North Africa using the hidden Markov model and regularized autoregressive models. J Defense Model Simul.

[78] Wu C, Gerber MS (2017). Forecasting civil unrest using social media and protest participation theory. IEEE Trans Comput Soc Syst.

[79] Qiao F, Li P, Zhang X, Ding Z, Cheng J, Wang H (2017). Predicting social unrest events with hidden Markov models using GDELT. Discrete Dyn Nat Soc.

[80] Galla D, Burke J (2018). Predicting social unrest using GDELT. International conference on machine learning and data mining in pattern recognition.

[81] Alsaqabi A, Aldhubayi F, Albahli S (2019). Using machine learning for prediction of factors affecting crimes in Saudi Arabia. Proc. of the 2019 international conference on big data engineering.

[82] Joshi D, Basnet S, Arunachalam H, Soh L-K, Samal A, Ratcliff S, Werum R (2017). SURGE: social unrest reconnaissance GazEteer. Proc. of the 25th ACM SIGSPATIAL international conference on advances in geographic information systems.

[83] Fengcai Q, Jinsheng D, Li W (2020). An online framework for temporal social unrest event prediction using news stream. 2020 international conference on cyber-enabled distributed computing and knowledge discovery (CyberC).

[84] Qiao F, Li P, Deng J, Ding Z, Wang H (2015). Graph-based method for detecting occupy protest events using GDELT dataset. 2015 international conference on cyber-enabled distributed computing and knowledge discovery.

[85] González M, Alférez GH (2020) Application of data science to discover violence-related issues in Iraq. arXiv preprint. arXiv:2006.07980

[86] Keneshloo Y, Cadena J, Korkmaz G, Ramakrishnan N (2014). Detecting and forecasting domestic political crises: a graph-based approach. Proc. of the 2014 ACM conference on web science.

[87] Keertipati S, Savarimuthu BTR, Purvis M, Purvis M (2014). Multi-level analysis of peace and conflict data in GDELT. Proc. of the MLSDA 2014 2nd workshop on machine learning for sensory data analysis.

[88] Yonamine JE (2013) Predicting future levels of violence in Afghanistan districts using GDELT. Unpublished manuscript

[89] Yuan L, Song C, Cheng C, Shen S, Chen X, Wang Y (2020). The cooperative and conflictual interactions between the United States, Russia, and China: a quantitative analysis of event data. J Geogr Sci.

[90] Boecking B, Hall M, Schneider J (2015). Event prediction with learning algorithms—a study of events surrounding the Egyptian revolution of 2011 on the basis of micro blog data. Policy Internet.

[91] Ahmed MN, Barlacchi G, Braghin S, Calabrese F, Ferretti M, Lonij V, Nair R, Novack R, Paraszczak J, Toor AS (2016). A multi-scale approach to data-driven mass migration analysis. SoGood@ ECML-PKDD.

[92] Beine M, Bertinelli L, Cömertpay R, Litina A, Maystadt J-F, Zou B (2019). Refugee mobility: evidence from phone data in Turkey. Guide to mobile data analytics in refugee scenarios.

[93] Bertoli S, Cintia P, Giannotti F, Madinier E, Ozden C, Packard M, Pedreschi D, Rapoport H, Sîrbu A, Speciale B (2019). Integration of Syrian refugees: insights from D4R, media events and housing market data. Guide to mobile data analytics in refugee scenarios.

[94] Carammia M, Iacus SM, Wilkin T (2020) Forecasting asylum applications in the European union with machine learning and data at scale. arXiv preprint. arXiv:2011.04348 10.1038/s41598-022-05241-8 PMC8795256 35087096

[95] Voukelatou V, Miliou I, Giannotti F, Pappalardo L (2021). Code release for EPJ paper. Zenodo.

[96] Best RH, Carpino C, Crescenzi MJ (2013). An analysis of the TABARI coding system. Confl Manage Peace Sci.

[97] Schrodt PA (2012) Cameo: conflict and mediation event observations event and actor codebook. Pennsylvania State University

[98] Leetaru K, Schrodt PA (2013). Gdelt: global data on events, location, and tone, 1979–2012. ISA annual convention.

[99] Fernandes S, Bernardino J (2015). What is bigquery?. Proceedings of the 19th international database engineering & applications symposium. IDEAS ’15.

[100] Hyndman RJ, Athanasopoulos G (2018). Forecasting: principles and practice.

[101] Zeller TL, Metzger LM (2013). Good bye traditional budgeting, hello rolling forecast: has the time come?. Am J Bus Educ.

[102] Schrodt PA (2011). Forecasting political conflict in Asia using latent Dirichlet allocation models. Annual meeting of the European political science association.

[103] Štrumbelj E, Kononenko I (2014). Explaining prediction models and individual predictions with feature contributions. Knowl Inf Syst.

[104] Ribeiro MT, Singh S, Guestrin C (2016). “Why should I trust you?” Explaining the predictions of any classifier. Proceedings of the 22nd ACM SIGKDD international conference on knowledge discovery and data mining.

[105] Ribeiro MT, Singh S, Guestrin C (2018). Anchors: high-precision model-agnostic explanations. Proceedings of the AAAI conference on artificial intelligence.

[106] James G, Witten D, Hastie T, Tibshirani R (2013). An introduction to statistical learning.

[107] Kassambara A (2018). Machine learning essentials: practical guide in R.

[108] De Myttenaere A, Golden B, Le Grand B, Rossi F (2016). Mean absolute percentage error for regression models. Neurocomputing.

[109] Swanson DA (2015) On the relationship among values of the same summary measure of error when used across multiple characteristics at the same point in time: an examination of MALPE and MAPE. Rev Econ Finance 5(1)

[110] Akoglu H (2018). User’s guide to correlation coefficients. Turk J Emerg Med.

[111] Cooper AF, Thakur R (2013). The group of twenty (G20).

[112] Hollis ME, Downey S, del Carmen A, Dobbs RR (2017). The relationship between media portrayals and crime: perceptions of fear of crime among citizens. Crime Prev Community Saf.

[113] Dehghan A, Montgomery L, Arciniegas-Mendez M, Ferman-Guerra M Predicting news bias

[114] Colaresi M, Mahmood Z (2017). Do the robot: lessons from machine learning to improve conflict forecasting. J Peace Res.

[115] Hattotuwa S (2013). Big data and peacebuilding. Stab Int J Secur Dev.

[116] Iacus SM, Yoshida N (2018). Simulation and inference for stochastic processes with YUIMA. A comprehensive R framework for SDEs and other stochastic processes.

