# Supplementary material for: Understanding peace through the world news
Source: EPJ Data Sci. 2022 Jan 21;11(1):2. doi: 10.1140/epjds/s13688-022-00315-z (PMC8777429; doi:10.1140/epjds/s13688-022-00315-z)
Supplement: Supplementary file 1 — Supplementary information (PDF 6.1 MB) [file 13688_2022_315_MOESM1_ESM.pdf]

# Understanding peace through the world news
# Supplementary Material

Vasiliki Voukelatou, Ioanna Miliou, Fosca Giannotti, Luca Pappalardo

## I. SUPPLEMENTARY NOTE 1: INDICATORS OF GPI

The GPI is a composite index of these 23 indicators weighted and combined into one overall score. The GPI comprises 23 indicators of the absence of violence or fear of violence aggregated into three major categories: Ongoing Domestic & International Conflict, Societal Safety & Security, and Militarization:

- Ongoing Domestic & International Conflict includes: "Number and duration of internal conflicts", "Number of deaths from external organized conflict", "Number of deaths from internal organized conflict", "Number, duration and role in external conflicts", "Intensity of organized internal conflict", and "Relations with neighbouring countries".
- Societal Safety & Security encompasses: "Level of perceived criminality in society", "Number of refugees and internally displaced people as a percentage of the population" , "Political instability", "Political Terror Scale", "Impact of terrorism", "Number of homicides per 100,000 people", "Level of violent crime", "Likelihood of violent demonstrations", "Number of jailed population per 100,000 people", "Number of internal security officers, and police per 100,000 people".
- Militarization contains: "Military expenditure as a percentage of GDP", "Number of armed services personnel per 100,000 people", "Volume of transfers of major conventional weapons as recipient (imports) per 100,000 people", "Volume of transfers of major conventional weapons as supplier (exports) per 100,000 people", "Financial contribution to UN peacekeeping missions", "Nuclear and heavy weapons capabilities", and "Ease of access to small arms and light weapons".

## II. SUPPLEMENTARY NOTE 2: TOPICS OF GDELT

The GDELT event categories we use are related to 20 topics, as described below. For each topic, we provide a short description and a few examples of event categories:

Make Public Statement refers to public statements expressed verbally or in action, such as "Make statement", "Make pessimistic comment", and "Decline comment". Appeal refers to requests, proposals, suggestions and appeals, such as "Appeal for material cooperation", "Appeal for economic cooperation", and "Appeal to others to settle dispute". Express Intent To Cooperate refers to offer, promise, agree to, or otherwise indicate willingness or commitment to cooperate, such as "Express intent to engage in material cooperation" and "Express intent to provide material aid". Consult refers to consultations and meetings, such as "Discuss by telephone" and "Host a visit". Engage In Diplomatic Cooperation refers to initiate, resume, improve, or expand diplomatic, non-material cooperation or exchange, such as "Sign formal agreement" and "Praise or endorse". Engage In Material Cooperation refers to initiate, resume, improve, or expand material cooperation or exchange, such as "Cooperate economically" and "Share intelligence or information". Provide Aid refers to provisions and extension of material aid, such as "Provide economic aid" and "Provide humanitarian aid". Yield refers to yieldings and concessions, such as "Accede to requests or demands for political reform", "De-escalate military engagement", and "Return, release". Investigate refers to non-covert investigations, such as "Investigate crime, corruption" and "Investigate human rights abuses". Demand refers to demands and orders, such as "Demand political reform" and "Demand settling of dispute". Disapprove refers to the expression of disapprovals, objections, and complaints, such as "Criticize or denounce" and "Complain officially". Reject refers to rejections and refusals, such as "Reject request or demand for material aid" and "Reject mediation". Threaten refers to threats, coercive or forceful warnings with serious potential repercussions, such as "Threaten with military force" and "Threaten with administrative sanctions". Protest refers to civilian demonstrations and other collective actions carried out as protests such as "Demonstrate or rally" and "Conduct strike or boycott". Exhibit Force Posture refers to military or police moves that fall short of the actual use of force, such as "Exhibit military or police power" and "Increase military alert status". Reduce Relations refers to reductions in normal, routine, or cooperative relations, such as "Reduce or break diplomatic relations" and "Halt negotiations". Coerce refers to repression, violence against civilians, or their rights or properties, such as "Arrest, detain" and "Seize or damage property". Assault refers to the use of different forms of violence, such as

"Conduct non-military bombing" and "Abduct, hijack, take hostage". FIGHT refers to uses of conventional force and acts of war, such as "Use conventional military force" and "Fight with small arms and light weapons". ENGAGE IN UNCONVENTIONAL MASS VIOLENCE refers to uses of unconventional force that are meant to cause mass destruction, casualties, and suffering, such as "Engage in ethnic cleansing" and "Detonate nuclear weapons".

## III. SUPPLEMENTARY NOTE 3: MACHINE LEARNING MODELS

### *Linear regression*

Linear regression, one of the simplest and most widely used regression techniques, calculates the estimators of the regression coefficients (the predicted weights) by minimizing the sum of squared residuals [1]. One of its main advantages is the ease of interpreting results.

### *Elastic Net*

Elastic Net is a regularized variable selection regression method. One of the essential advantages of Elastic Net is that it combines penalization techniques from the Lasso and Ridge regression methods into a single algorithm [2]. Lasso regression penalizes the sum of absolute values of the coefficients (L1 penalty), Ridge regression penalizes the sum of squared coefficients (L2 penalty), while Elastic Net imposes both L1 and L2 penalties. This means that Elastic Net can completely remove weak variables, as Lasso does, or reduce them by bringing them closer to zero, as Ridge does. Therefore, it does not lose valuable information, but still imposes penalties to lessen the impact of certain variables.

### *Decision Tree*

Decision trees are used to visually and explicitly represent decisions, in the form of a tree structure. A decision tree is called regression tree when the dependent variable takes continuous values [2]. The goal of using a regression tree is to create a training model that can predict the value of the dependent variable by learning simple decision rules inferred from the training data. The regression tree induction algorithm divides the dataset into smaller data groups, while simultaneously an associated decision tree is incrementally developed. The final tree consists of decision nodes and leaf nodes. A decision node has two or more branches, each representing values for the variable tested. A leaf node represents a decision on the value of the dependent variable. The topmost decision node, called the root node, corresponds to the most important variable. The main difference between a regression tree and a decision tree is that for regression problems, the objective function is to minimize the variance in each partition.

### *Support Vector Regression (SVR)*

SVR [3] is a regression learning approach which, comparing to other regression algorithms that try to minimize the error between the real and predicted value, uses a symmetrical loss function that equally penalizes high and low misestimates. In particular, it forms a tube symmetrically around the estimated function (hyperplane), such that the absolute values of errors less than a certain threshold are penalised both above and below the estimate, but those within the threshold do not receive any penalty. The most commonly used kernels, for finding the hyperplane, is the Radial Basis Function (RBF) kernel, that we also use for our analysis. One of the main advantages of SVR is that its computational complexity does not depend on the dimensionality of the input space. Moreover, it has excellent generalization capability, and provides high prediction accuracy.

### *Random Forest*

Random Forest limits the risk of a Decision Tree to overfit the training data [2]. As the names "Tree" and "Forest" imply, a Random Regression Forest is essentially a collection of individual Regression Trees that operate as a whole. A Regression Tree is built on the entire dataset, using all the variables of interest. On the contrary, Random Forest builds multiple Regression Trees from randomly selecting observations and specific variables and then averages over

all trees' prediction. Individually, predictions made by Regression Trees may not be accurate, but combined, are, on average, closer to the true value.

### *Extreme Gradient Boosting (XGBoost)*

XGBoost [4] is a scalable machine learning regression system for tree boosting. It uses a gradient descent algorithm and incorporates a regularized model to prevent overfitting. Comparing to Random Forest that builds each tree independently and combines them in parallel, XGBoost uses boosting, combining weak learners (usually decision trees with only one split, called decision stumps) sequentially, so that each new tree corrects the errors of the previous one. In particular, XGBoost corrects the previous mistakes made by the model, learns from it and its next step enhances the performance until there is no scope of further improvements. Its main advantage is that it is fast to execute and gives high accuracy.

## IV. SUPPLEMENTARY NOTE 4: HYPERPARAMETERS

The hyperparameters we tune for Elastic Net are $\alpha$, which is the relative importance of the L1 (LASSO) and L2 (Ridge) penalties, and $\lambda$, which is the amount of regularization used in the model. For Decision Tree, we tune the complexity parameters $maxdepth$, which is the maximum depth of the tree), $minsamplessplit$, which is the minimum number of samples required to split an internal node, and $minsamplesleaf$, which is the minimum number of samples required to be at a leaf node. For Random Forest, similarly to Decision Tree, we tune the $maxdepth$, the $minsamplessplit$, and the $minsamplesleaf$. We also tune the $nestimators$, which accounts for the number of number of trees in the model, and the $maxfeatures$, which corresponds to the number of variables to consider when looking for the best split. For XGBoost, we tune the $nestimators$, similarly to Random Forest, and the $maxdepth$, similarly to Decision Tree. We also tune the $learningrate$, a value that in each boosting step, shrinks the weight of new variables, preventing overfitting or a local minimum, and $colsample_bytree$, which represents the fraction of columns to be subsampled, it is related to the speed of the algorithm and it prevents overfitting. Last, for SVR RBF model we tune the regularization parameter $C$, which imposes a penalty to the model for making an error, and $gamma$ parameter, which defines how far the influence of a single training example reaches.

## V. SUPPLEMENTARY NOTE 5: PERFORMANCE INDICATORS

We consider the following indicators to assess the performance of the prediction models with respect to the ground-truth GPI values. Our notation is as follows: $y_t$ denotes the observed value of the GPI at time $t$, $x_t$ denotes the predicted value by the model at time $t$, $\bar{y}$ denotes the mean or average of the values $y_t$ and similarly $\bar{x}$ denotes the mean or average of the values $x_t$.
***Pearson Correlation***, a measure of the linear dependence between two variables during a time period $[t_1, t_n]$, is defined as:

$$r = \frac{\sum_{t=1}^{n}(y_t - \bar{y})(x_t - \bar{x})}{\sqrt{\sum_{t=1}^{n}(y_t - \bar{y})^2}\sqrt{\sum_{t=1}^{n}(x_t - \bar{x})^2}} \ . \tag{1}$$

***Root Mean Square Error (RMSE)***, a measure of prediction accuracy that represents the square root of the second sample moment of the differences between predicted values and actual values, is defined as:

$$RMSE = \sqrt{\frac{1}{n}\sum_{t=1}^{n}(x_t - y_t)^2} \ . \tag{2}$$

***Mean Absolute Percentage Error (MAPE)***, a measure of prediction accuracy between predicted and true values, is defined as:

$$MAPE = \left(\frac{1}{n}\sum_{t=1}^{n}|\frac{y_t - x_t}{y_t}|\right) \times 100 \ . \tag{3}$$

## VI. SUPPLEMENTARY NOTE 6: LINEAR MODELS RESULTS

The median Pearson Correlation for the Linear models for the 1-month-ahead predictions is 0.069, and the median MAPE is 39.273. These results demonstrate that Linear models show lower performance not only from the XGBoost models (0.521, and 1.593, respectively), but also from the Elastic Net models (0.327, and 1.997, respectively), already from the 1-month-ahead predictions.

## VII. SUPPLEMENTARY NOTE 7: RMSE RESULTS

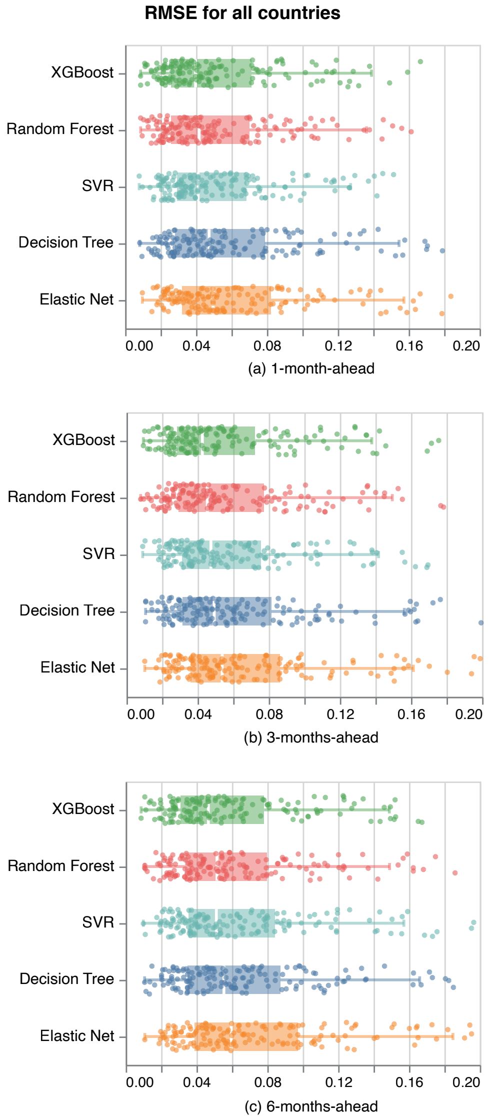


FIG. 1: **RMSE for all country models.** RMSE between the real and the predicted 1-, 3-, and 6-months-ahead GPI values at a country level, for all prediction models. The boxplots represent the distribution of the aforementioned performance indicators for all country models. The plots' data points correspond to each country model. Overall, XGBoost models outperform the rest of the four models.

## VIII. SUPPLEMENTARY NOTE 8: SCATTER PLOTS OF THE REAL AND ESTIMATED GPI VALUES

Figure 2 compares the real and estimated GPI values, showing a strong linear relationship between the two. In particular, Figure 2a presents the scatter plot of the real and predicted GPI values of all the countries, while Figures 2b-d focus on the corresponding values of Iceland, Saudi Arabia, and Pakistan. These countries indicate that the models show high performance for low, medium, or high GPI values.

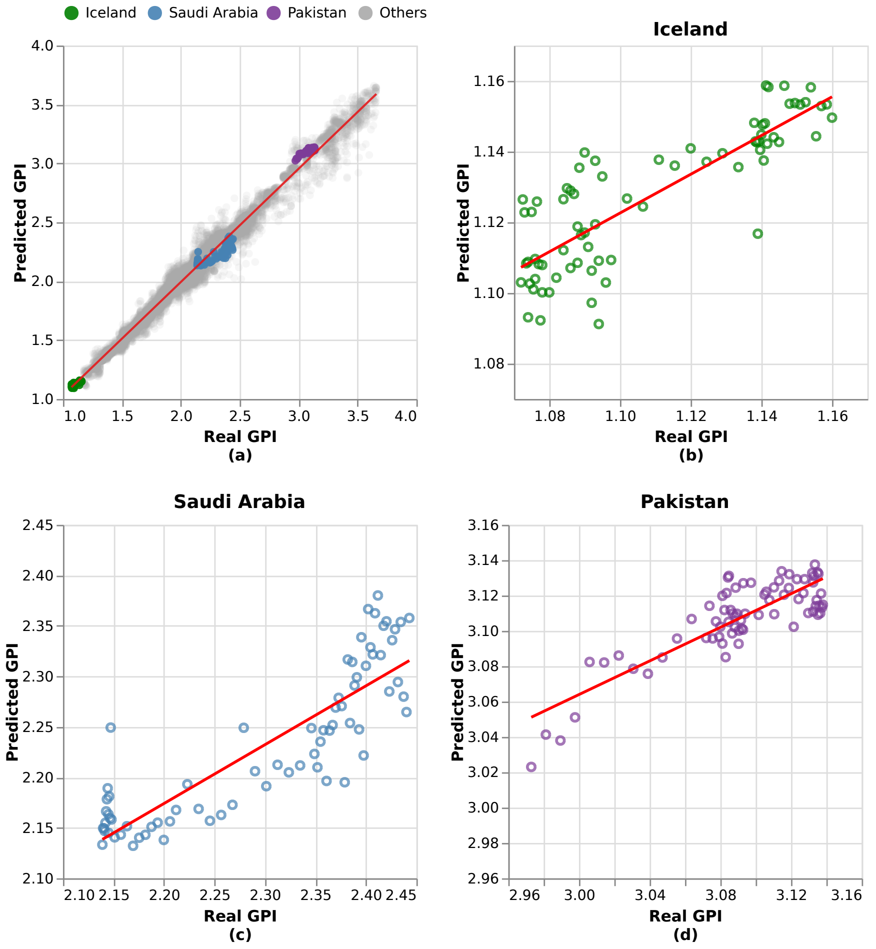


FIG. 2: **Scatter plots of the real and estimated GPI values.** (a) Scatter plots of the real and estimated GPI values for all country models. (b-d) Real versus estimate GPI values for Iceland (b), Saudi Arabia (c), and Pakistan (d).


[1] James, G., Witten, D., Hastie, T., Tibshirani, R.: An Introduction to Statistical Learning vol. 112. Springer, (2013)

[2] Hastie, T., Tibshirani, R., Friedman, J.: The Elements of Statistical Learning: Data Mining, Inference, and Prediction. Springer, (2009)

[3] Awad, M., Khanna, R.: Support vector regression. In: Efficient Learning Machines, pp. 67–80. Springer, (2015)

[4] Chen, T., Guestrin, C.: Xgboost: A scalable tree boosting system. In: Proceedings of the 22nd Acm Sigkdd International Conference on Knowledge Discovery and Data Mining, pp. 785–794 (2016)
